# Intrinsic capacity and its associations with incident dependence and mortality in 10/66 Dementia Research Group studies in Latin America, India, and China: A population-based cohort study

**DOI:** 10.1371/journal.pmed.1003097

**Published:** 2021-09-14

**Authors:** Martin J. Prince, Daisy Acosta, Mariella Guerra, Yueqin Huang, K. S. Jacob, Ivonne Z. Jimenez-Velazquez, A. T. Jotheeswaran, Juan J. Llibre Rodriguez, Aquiles Salas, Ana Luisa Sosa, Isaac Acosta, Rosie Mayston, Zhaorui Liu, Jorge J. Llibre-Guerra, A. Matthew Prina, Adolfo Valhuerdi

**Affiliations:** 1 King’s Global Health Institute, King’s College London, London, United Kingdom; 2 Health Service and Population Research Department, Institute of Psychiatry, Psychology and Neuroscience, King’s College London, London, United Kingdom; 3 Geriatric Section, Internal Medicine Department, Universidad Nacional Pedro Henríquez Ureña, Santo Domingo, Dominican Republic; 4 Psychogeriatric Unit, National Institute of Mental Health “Honorio Delgado Hideyo Noguchi”, Lima, Peru; 5 Centro de la Memoria y Desordenes Relacionados, Lima, Peru; 6 Institute of Mental Health, Peking University, Beijing, China; 7 Christian Medical College and Hospital, Vellore, India; 8 Geriatrics Program, Internal Medicine Department, School of Medicine, University of Puerto Rico, San Juan, Puerto Rico; 9 World Health Organization, Geneva, Switzerland; 10 Facultad de Medicina Finlay-Albarrán, Medical University of Havana, Havana, Cuba; 11 Medicine Department, Caracas University Hospital, Caracas, Venezuela; 12 Faculty of Medicine, Universidad Central de Venezuela, Caracas, Venezuela; 13 Laboratory of the Dementias, National Institute of Neurology and Neurosurgery of Mexico, Autonomous National University of Mexico, Mexico City, Mexico; 14 Department of Global Health and Social Medicine, King’s College London, London, United Kingdom; 15 Institute of Neurology “Dr. Rafael Estrada”, Havana, Cuba; 16 Universidad de Ciencias Médicas de Matanzas, Matanzas, Cuba; University of New South Wales, AUSTRALIA

## Abstract

**Background:**

The World Health Organization (WHO) has reframed health and healthcare for older people around achieving the goal of healthy ageing. The recent WHO Integrated Care for Older People (ICOPE) guidelines focus on maintaining intrinsic capacity, i.e., addressing declines in neuromusculoskeletal, vitality, sensory, cognitive, psychological, and continence domains, aiming to prevent or delay the onset of dependence. The target group with 1 or more declines in intrinsic capacity (DICs) is broad, and implementation may be challenging in less-resourced settings. We aimed to inform planning by assessing intrinsic capacity prevalence, by characterising the target group, and by validating the general approach—testing hypotheses that DIC was consistently associated with higher risks of incident dependence and death.

**Methods and findings:**

We conducted population-based cohort studies (baseline, 2003–2007) in urban sites in Cuba, Dominican Republic, Puerto Rico, and Venezuela, and rural and urban sites in Peru, Mexico, India, and China. Door-knocking identified eligible participants, aged 65 years and over and normally resident in each geographically defined catchment area. Sociodemographic, behaviour and lifestyle, health, and healthcare utilisation and cost questionnaires, and physical assessments were administered to all participants, with incident dependence and mortality ascertained 3 to 5 years later (2008–2010). In 12 sites in 8 countries, 17,031 participants were surveyed at baseline. Overall mean age was 74.2 years, range of means by site 71.3–76.3 years; 62.4% were female, range 53.4%–67.3%. At baseline, only 30% retained full capacity across all domains. The proportion retaining capacity fell sharply with increasing age, and declines affecting multiple domains were more common. Poverty, morbidity (particularly dementia, depression, and stroke), and disability were concentrated among those with DIC, although only 10% were frail, and a further 9% had needs for care. Hypertension and lifestyle risk factors for chronic disease, and healthcare utilisation and costs, were more evenly distributed in the population. In total, 15,901 participants were included in the mortality cohort (2,602 deaths/53,911 person-years of follow-up), and 12,939 participants in the dependence cohort (1,896 incident cases/38,320 person-years). One or more DICs strongly and independently predicted incident dependence (pooled adjusted subhazard ratio 1.91, 95% CI 1.69–2.17) and death (pooled adjusted hazard ratio 1.66, 95% CI 1.49–1.85). Relative risks were higher for those who were frail, but were also substantially elevated for the much larger sub-groups yet to become frail. Mortality was mainly concentrated in the frail and dependent sub-groups. The main limitations were potential for DIC exposure misclassification and attrition bias.

**Conclusions:**

In this study we observed a high prevalence of DICs, particularly in older age groups. Those affected had substantially increased risks of dependence and death. Most needs for care arose in those with DIC yet to become frail. Our findings provide some support for the strategy of optimising intrinsic capacity in pursuit of healthy ageing. Implementation at scale requires community-based screening and assessment, and a stepped-care intervention approach, with redefined roles for community healthcare workers and efforts to engage, train, and support them in these tasks. ICOPE might be usefully integrated into community programmes for detecting and case managing chronic diseases including hypertension and diabetes.

## Abstract 2

In a population-based cohort study, Martin J. Prince and colleagues evaluate intrinsic capacity and its associations with incident dependence and mortality in Latin America, India and China.

## Author summary

**Why was this study done?:**

- As we grow older, we are affected to different degrees by declines in the working of our organs and body systems, limiting our ability to get around, be well nourished, see, hear, and think clearly, stay continent, and maintain mood and well-being.
- The World Health Organization has proposed a programme—Integrated Care for Older People (ICOPE)—supported by evidence that simple interventions may slow or reverse these processes, with potential to improve health and functioning and delay needs for care developing among older people.
- ICOPE targets low- and middle-income countries, but we do not yet know how many older people in these settings are significantly affected by early age-related decline, or whether these changes predict who will go on to deteriorate further, leading to needs for care and earlier death.

**What did the researchers do and find?:**

- We had already completed (between 2003 and 2007) baseline community surveys of 17,031 people aged 65 years and over living in 12 urban and rural sites in 6 Latin American countries, India, and China; we then tried to interview them again 3 to 5 years later (between 2008 and 2010) to identify who had died (in 11 sites, among 15,901 older people) and who had developed needs for care (in 10 sites, among 12,939 older people).
- Analysing baseline data from these studies, we found that between two-thirds and three-quarters of older people were affected by significant decline in at least 1 of the areas targeted by the ICOPE programme; this proportion was highest among the older old, who were also more likely to have problems in multiple areas.
- Most (nearly three-quarters) of those with significant decline at baseline had not yet gone on to become physically frail or develop needs for care; they were nevertheless 1.7 to 1.9 times more likely than those without any significant decline to develop needs for care over the follow-up period, and 1.3 to 1.4 times more likely to have died.

**What do these findings mean?:**

- Delivering comprehensive assessment, care planning, and community interventions to up to three-quarters of the older population would be a major challenge for poorly resourced health systems in low- and middle-income countries.
- Nevertheless, findings from our analyses support the World Health Organization’s strategy to promote health and well-being by targeting a broad group with early decline who do seem to be at increased risk of adverse outcomes.
- Future research should focus upon feasible, acceptable, and efficient methods of delivering the ICOPE programme (implementation science) and its effectiveness and cost-effectiveness in improving health and social outcomes (controlled trials).
- The main limitations were that health declines were measured in different ways, some of which may have been imprecise, and those who could not be followed up may have had different characteristics to those who were included in the analysis.

## Introduction

In May 2018, the World Health Organization (WHO) announced a new set of priorities—3 ‘triple billion’ targets to ensure that by 2023, 1 billion more people benefit from universal health coverage, 1 billion more people have better protection from health emergencies, and 1 billion more people enjoy better health and well-being. Older people, who by 2019 will number 1 billion, typically experience worse health and health-related quality of life than younger persons yet do not access healthcare services proportionately; they are particularly vulnerable in emergency situations and account for half or more of all deaths after natural disasters [1]. These disadvantages are most pronounced in low- and middle-income countries (LMICs). Older peoples’ needs therefore need to be accorded more priority. The WHO has also been active on this front, launching the landmark 2015 World Report on Ageing and Health [1], the Global Strategy and Action Plan on Ageing and Health (2016–2020) [2], and, in 2017, the evidence-based Integrated Care for Older People (ICOPE) guidelines [3,4].

The WHO World Report reframes health and healthcare for older people around the goal of healthy ageing, achieved when functional abilities, the ‘health-related attributes that enable people to be and do what they have reason to value’, are developed and maintained across the life course [1]. At the core of functional ability are the physical, mental, and cognitive intrinsic capacities of the individual, interacting with their environment. The ICOPE guidelines support the WHO Global Strategy core objective of ‘aligning health systems to the needs of older populations’. Implementation will promote community-level attention to declines in intrinsic capacity (DICs) among older adults. Thirteen recommendations cover mobility loss, malnutrition, visual impairment and hearing loss, cognitive impairment, and depressive symptoms, with additional modules on geriatric syndromes (urinary incontinence and risk of falls) and support for carers [3,4]. WHO envisages brief screening for intrinsic capacity. Significant declines would prompt more comprehensive needs assessment, but the prevalence and distribution of DICs have yet to be established in LMICs. The emphasis is upon bringing assessment and health and social care to the older person: in primary care, at home, and in the community. Of necessity, this involves task-shifting and task-sharing, particularly in low-resourced settings where specialist older care services are underdeveloped. Given the extent of unmet needs, the optimal targeting of the ICOPE intervention will be an important consideration, if health gains are to be realised in a cost-effective manner.

Aside from the evidence base that supports each of the 13 ICOPE recommendations, the broad approach of multidimensional assessment and management is strongly supported, mainly by evidence from high-income countries (HICs). The disability cascade is neither inevitable, unidirectional, nor irreversible. Longitudinal studies of frailty in older populations show an overall tendency for stability and modest net progression, with little reversion from frail to completely robust states; however, significant numbers of individuals do improve, transiting from pre-frail to robust, and from frail to pre-frail states [5–10]. Findings are similar from studies of transitions into and out of states of dependence [11–14], with some suggestion of less stability and more reversion in LMICs [12,13]. More favourable trajectories are associated with more education [10,11,15], higher socioeconomic status [15], and adherence to healthy behaviours and lifestyles, particularly physical activity [14,16]. Certain chronic progressive diseases (diabetes, cancer, chronic obstructive pulmonary disease [COPD], osteoarthritis, dementia, and cerebrovascular disease) are associated with a worse prognosis [6,17], and may mediate much of the effect of education [17]. The critical time window for interventions, if any, has not yet been clearly established. A systematic review in 2008 identified 89 trials of home-based multi-dimensional assessment and intervention, all conducted in HICs [18]. While, overall, there was evidence for improvements in physical function, fewer falls, and fewer admissions to nursing homes and hospitals, these benefits were mainly confined to interventions in the general older adult population, rather than amongst those selected because they were frail. This may have contributed to a widely held perception that early intervention is better, even necessary, to prevent the onset of frailty and disability. However, subsequent trials in the Netherlands [19], Australia [20], and the US [21] also indicate potential for improvements in health, functioning, and quality of life among frail and dependent older people, with some evidence to support the cost-effectiveness of multi-dimensional interventions [20,22].

To inform planning of ICOPE implementation activities we conducted a secondary analysis of data collected from the 10/66 Dementia Research Group (10/66 DRG) population-based baseline and incidence wave surveys in 12 catchment areas in 8 LMICs in Latin America, China, and India. Our objectives were to (1) determine the prevalence of intrinsic capacity overall, and in its several domains, clarifying the proportion of individuals likely to be identified with significant DIC, and hence to be the target population for the ICOPE intervention; (2) characterise the target group, and compare them with others, for burden of disease (behavioural and lifestyle risk factors, morbidities, and disability) and use of healthcare services; (3) establish the extent of the overlap of DIC with frailty and dependence (needs for care), and the concentration of burden indicators in each of these 3 groups when defined hierarchically; and (4) clarify the prospective association of DIC with incident dependence and mortality.

## Methods

This study is reported as per the Strengthening the Reporting of Observational Studies in Epidemiology (STROBE) guideline (S1 Checklist).

### Settings and study design

The 10/66 DRG population-based studies of ageing and dementia in LMICs comprised baseline surveys of older people aged 65 years and over living in geographically defined catchment areas in 8 countries, with a follow-up 3 to 5 years later. The study protocols [23] and the cohort profile (with detailed information on catchment area selection, sample size determination, cohort resource, and follow-up) [24] are documented in open-access publications. The current analyses focus upon urban and rural sites in Peru (urban Lima and rural Canete), Mexico (urban Mexico City and rural Morelos state), China (urban Xicheng and rural Daxing), and India (urban Chennai and rural Vellore), and urban sites in Cuba (Havana/Matanzas), Dominican Republic (Santo Domingo), Puerto Rico (Bayamon), and Venezuela (Caracas). The cohort was defined at baseline by systematic door-knocking of all households in each catchment area, to identify those eligible, who were all those normally resident and aged 65 years or over on the defined census date. Baseline population-based surveys were carried out between 2003 and 2007, and incidence wave follow-up assessments between 2008 and 2010 [24]. For India, the follow-up was conducted only in the urban site and comprised a mortality sweep only. The protocols for the 1-phase surveys comprised a clinical interview; a health, medical history, healthcare utilisation, and lifestyle interview; a cognitive assessment; a physical examination; and an informant interview. All participants were interviewed at home, unless they requested an alternative arrangement. The study protocol and the consent procedures were approved by the King’s College London research ethics committee and in all countries where the research was carried out: (1) Medical Ethics Committee of Peking University Sixth Hospital (Institute of Mental Health, China); (2) Memory Institute and Related Disorders (IMEDER) Ethics Committee (Peru); (3) Finlay-Albarrán Medical Faculty of Havana Medical University Ethical Committee (Cuba); (4) Hospital Universitario de Caracas Ethics Committee (Venezuela); (5) Consejo Nacional de Bioética y Salud (Dominican Republic); (6) Instituto Nacional de Neurología y Neurocirugía Ethics Committee (Mexico); (7) University of Puerto Rico Medical Sciences Campus Institutional Review Board; (8) Christian Medical College Research and Ethics Committee (Vellore, India); and (9) Voluntary Health Services Multi Speciality Hospital and Research Centre Institutional Ethics Committee (Chennai, India). Informed consent was documented in writing in all cases. Literate participants signed their consent. For participants who were illiterate, the information sheet was read to them in the presence of a literate independent witness, who attested by signature that this process was completed, and that the participant had provided informed consent. For participants who lacked capacity to consent, agreement for their participation was obtained from next of kin. These procedures were approved by the ethics committees.

### Measures

Full details are provided in the published study protocol [23], and the complete survey questionnaires are available online at https://www.alz.co.uk/1066/population_based_study_prevalence.php (baseline prevalence wave questionnaires in English, Spanish, and Tamil) and https://www.alz.co.uk/1066/population_based_study_incidence.php (incidence wave questionnaires in English and Spanish). Here we summarise the measures directly relevant to the analyses presented in this paper.

Age was ascertained from participant and informant reports, documented age, or an event calendar. Education level was self-reported, and coded as no education, did not complete primary, completed primary, secondary, or tertiary education. Food insecurity was defined as reporting going hungry in the last 1 month, due to inadequate resources to procure food.

#### Intrinsic capacities

Seven intrinsic capacities were evaluated at baseline, linked to the concepts and, where possible, the operationalisations currently within the WHO ICOPE draft guidance on comprehensive assessment. For each intrinsic capacity we applied a threshold to determine whether the individual has retained capacity or experienced DIC. It is important to note that the decline is inferred from a single assessment, and not directly observed. As such, long-term stable impairment could have been misclassified as age-related decline. The intrinsic capacities are as follows. (1) Neuromusculoskeletal capacity: Walking speed was assessed using a timed walking test (5 metres at usual speed, turn, and return to the starting point); those completing the test in less than 16 seconds were considered to have capacity (allowing 3 seconds to make the turn, this corresponds to a walking speed of >0.8 m/s). (2) Vitality: Nutrition was considered adequate if the older person did not report weight loss of ≥4.5 kg in the last 3 months, and if their mid-upper arm circumference was measured to be ≥22 cm; this cut-point is used in the Mini Nutritional Assessment (MNA) to identify the most severe level of undernutrition [25]. (3) Sensory capacity (visual impairment): Vision was considered adequate when the older person did not report ‘eyesight problems’ that interfered with their activities to at least some extent, and when they were not identified by the interviewer as being functionally blind. (4) Sensory capacity (hearing loss): Hearing was considered adequate when the older person did not report ‘hearing problems or deafness’ that interfered with their activities to at least some extent, and when they were not identified by the interviewer as being profoundly deaf. (5) Cognitive capacity: Cognitive function was assessed using the Community Screening Instrument for Dementia (CSI-D) COGSCORE, which tests multiple domains of cognitive function and has been found to have robust cross-cultural measurement properties in the 10/66 DRG study sites; those who scored ≥29.5 were considered to have cognitive capacity, with scores below that threshold identifying ‘probable dementia’ [26]. (6) Psychological capacity: Psychological capacity was considered to be present if participants endorsed 3 or fewer of the 12 depression symptoms covered in the EURO-D depression scale; in previous analyses this cut-point identified individuals with significant impairment of health-related quality of life [27], although a higher cut-point is better for identifying clinical cases [28]. (7) Continence: Incontinence (urinary, faecal, or both) was established from informant report only, and capacity was maintained if none of these were reported. Continence is not currently a primary focus of the ICOPE comprehensive assessment tool, although guidelines for the assessment and management of incontinence have been prepared by the guideline development group.

#### Frailty

A proposed physical frailty phenotype [29] includes 5 frailty indicators (exhaustion, weight loss, weak grip strength, slow walking speed, and low energy expenditure). Individuals are frail if they meet 3 or more of the 5 criteria, pre-frail if they meet 1 or 2, and non-frail if they meet none of the 5 criteria [29]. We assessed 4 of the 5 indicators of frailty, omitting hand grip strength and using slightly different operationalisations to those originally proposed for exhaustion, weight loss, and energy consumption [30]. As handgrip strength was not measured, we considered participants frail if they fulfilled criteria for 2 or more of the 4 frailty indicators, and pre-frail if they met 1; the effect is the same as imputing a value of 1 for handgrip strength. For most analyses we allocate frail or pre-frail individuals who already need care to the dependent sub-group, consistent with the concept of frailty as a vulnerability for disability and dependence.

#### Behavioural and lifestyle risk factors

Lifetime smoking history, current physical activity, alcohol consumption, and dietary intake of fruit and vegetables were ascertained from self-report. Adverse behaviours were defined as current smoking, self-report of being ‘not very’ or ‘not at all’ physically active, hazardous alcohol use (weekly consumption of ≥28 units of alcohol for men and ≥21 units for women), and fewer than 4 portions of fruit or vegetable in the last 3 days. Waist circumference was measured in centimetres using a flexible tape measure; central obesity was defined according to the criteria for metabolic syndrome in the Third Report of the National Cholesterol Education Program—a waist circumference of more than 101.6 centimetres in men and of more than 88.9 centimetres in women.

#### Morbidity

We assessed physical, mental, and cognitive morbidity through measures of hypertension and diabetes, and the main contributors to disability and dependence [31,32]: stroke, dementia, and depression. Dementia was diagnosed according to the cross-culturally developed, calibrated, and validated 10/66 DRG dementia diagnosis algorithm [26]. ICD-10 depressive episode was diagnosed using a computerised algorithm applied to the GMS structured clinical interview [33]. Stroke was self-reported, but confirmed by the interviewer as having characteristic symptoms lasting for more than 24 hours [34]. Hypertension was ascertained through blood pressure measurement, applying WHO/International Society of Hypertension criteria (SBP ≥ 140 mm Hg and/or DBP ≥ 90 mm Hg), and self-report of previous diagnosis and treatment; those with elevated blood pressure were considered to have uncontrolled hypertension, regardless of detection or treatment [35].

#### Disability and dependence

Disability was assessed using the WHODAS 2.0 scale, developed by the WHO as a culture-fair assessment tool for use in cross-cultural comparative epidemiological and health services research [36,37]. Dependence (needs for care) was interviewer-rated after a series of open-ended probing questions administered towards the end of the assessment to a key informant, followed, as appropriate, by a detailed assessment of caregiving input and roles [32]. The rating of no needs for care, needs care some of the time, or needs care much of the time was made in the light of all information collected and observations made over the course of the assessment. Further supporting information has been provided on the assessment procedures, and the evidence for the construct validity of the dependence outcome (S1 Working Paper). To identify probable cases of incident dependence among participants who had died during the follow-up period, a predictive model for incident dependence was developed using variables from the informant section of the CSI-D informant interview, which was available for all participants. For deceased participants this was conducted as part of an informant verbal autopsy interview, and referred to the period before death. The model used age, the total CSI-D informant score, and 5 items from the CSI-D informant interview: activity, feeding, toileting, dressing, and household chores. The predictive model was developed from those who had survived, then applied to those who were deceased at follow-up to predict incident dependence [38].

#### Healthcare utilisation and costs

Details of healthcare cost estimations are provided elsewhere [39]. Participants were asked about contacts with primary healthcare professionals, public hospital doctors, other publicly provided professional healthcare services, and private healthcare services (private doctors, dentists, and traditional healers). For each service, participants were asked how often they had used it in the last 3 months, the duration of the consultation, and fees for the service. Travel costs were also elicited. Lengths of stay and out-of-pocket costs for hospital admissions, and total costs of medication paid out-of-pocket for any of these services, were also recorded. Out-of-pocket costs comprised the total annualised payments made by healthcare service users. Total costs from a public perspective reflect the actual cost to the provider, regardless of financing, including staff salaries, facilities and equipment, and overheads. Out-of-pocket costs and total costs were dichotomised at the 90th centile of the distribution in each site to reflect catastrophic healthcare spending and high total healthcare costs, respectively.

### Analysis

Although dependence and mortality were identified as outcomes of interest at the outset of our research programme, with general procedures for longitudinal analysis pre-specified and applied to publications to date (S1 Appendix) [38,40,41], our interest in DIC as an exposure stemmed from the publication of the WHO World Report on Ageing and Health [1] and work on the ICOPE guidelines [3,4]. The data analysis plan was therefore written well after the survey was designed and data collected.

We report, descriptively, the prevalence of retained intrinsic capacity for each of the 7 domains by site and, for the whole sample, prevalence by 5-year age group from 65–69 years to 90 years and over. Following suggestions from reviewers, site-specific prevalences were also directly standardised for age and gender; details of methods applied are provided in S2 Appendix. We describe the sociodemographic and health characteristics (lifestyle risk factors, morbidity, disability, and needs for care) and healthcare utilisation and costs of those with DIC affecting 1 or more domains, further stratified by frailty status and needs for care. Characteristics are compared with those of individuals with fully retained capacity (chi-squared tests and *t* tests), and across all 5 strata (full capacity, DIC only, DIC and pre-frail, DIC and frail, and DIC and dependent—chi squared tests and 1-way ANOVA tests for trend).

We modelled the effect of DIC exposures on the incidence of dependence (or ‘probable dependence’ among those who had died) using a competing risks regression derived from Fine and Gray’s proportional subhazards model [27] (Stata stcrreg command), based on a cumulative incidence function indicating the probability of failure (onset of dependence) before a given time, acknowledging the possibility of a competing event (dependence-free death), and reporting adjusted subhazard ratios (aSHRs). Competing risks regression keeps those who experience competing events at risk so that they can be counted as having no chance of failing. We modelled the effect of intrinsic capacity on mortality using Cox proportional hazards, generating cumulative survival probability curves and reporting adjusted hazard ratios (aHRs). Proportional hazards assumptions were checked using methods based on Schoenfeld residuals (Stata phtest command), and we fitted interactions with time for covariates that violated these assumptions. Time to death was the time from baseline interview to the exact date of death. Time to dependence onset (which could not be ascertained precisely) was the midpoint between baseline and follow-up interview/death. All effect sizes are presented with robust 95% confidence intervals adjusted for household clustering.

Models comprised (1) the effect of any DIC; (2) the effect of DIC when stratified as DIC only, DIC and pre-frail, DIC and frail, and (mortality model only) DIC and dependent; and (3) the effect of DIC per number of intrinsic capacity domains affected, before and after controlling for frailty (and dependence in the mortality model). All models were adjusted for age, gender, and education, and estimated separately for each site, with the results combined using a fixed effects meta-analysis. Higgins *I*^2^ estimates the proportion of between-site variability in the estimates accounted for by heterogeneity, as opposed to sampling error; up to 40% heterogeneity is conventionally considered negligible, while up to 60% reflects moderate heterogeneity [28].

Following reviewer suggestions, we carried out further analyses to describe attrition in the mortality and dependence cohorts in more detail, and to explore potential for bias by assessing the association of baseline demographic and health covariates with loss to follow-up (LTFU) (S2 Appendix).

## Results

### Sample characteristics

In all, 17,031 participants were surveyed at baseline in the 12 sites in 8 countries. Their characteristics have been reported in detail elsewhere [24]. Mean age varied between 71.3 and 76.3 years, and was higher in urban than rural areas and in more- than less-developed sites (Table 1). Most participants (62.4%) were female. Education levels varied widely among sites, with between 14.4% and 90.7% having completed primary education, and were lowest in rural sites in India, Mexico, and China and in the Dominican Republic, and highest in urban Peru, Puerto Rico, and Cuba. Food insecurity was commonest in urban (20.8%) and rural India (14.1%), rural Peru (13.5%), and the Dominican Republic (12.1%). Overall, 16.1% reported 3 or more physical impairments, 6.7% had a history of stroke, 5.5% met criteria for ICD-10 depressive episode in the last 1 month, and 9.3% for 10/66 dementia diagnosis. Physical impairments and stroke were less frequently reported in rural and less-developed sites, and depression was rarely identified in China.

**Table 1:** Baseline sample: Participants’ sociodemographic, socioeconomic, and health characteristics, by site.

Variable	Cuba	Dominican Republic	Puerto Rico	Peru urban	Peru rural	Venezuela	Mexico urban	Mexico rural	China urban	China rural	India urban	India rural
Response rate (%)	94	95	93	80	88	80	84	86	74	96	72	98
Achieved sample (*n*)	2,944	2,011	2,009	1,381	552	1,965	1,003	1,000	1,160	1,002	1,005	999
Age in years, mean (SD)	75.1 (7.0)	75.3 (7.5)	76.3 (7.4)	75.0 (7.4)	74.2 (7.3)	72.3 (6.9)	74.5 (6.6)	74.1 (6.7)	73.9 (6.2)	72.4 (6.0)	71.3 (6.1)	72.6 (5.8)
Female, *n* (%)	1,913/2,944 (65.0)	1,325/2,009 (65.9)	1,347/2,003 (67.3)	888/1,381 (64.3)	295/552 (53.4)	1,226/1,932 (63.5)	666/1,003 (66.4)	602/1,000 (60.2)	661/1,160 (57.0)	556/1,002 (55.5)	571/990 (57.7)	545/999 (54.6)
Education level, *n* (%)												
No education	75/2,936 (2.6)	392/1,992 (19.7)	72/1,999 (3.6)	37/1,373 (2.7)	84/544 (15.4)	156/1,925 (8.1)	227/1,001 (22.7)	327/1,000 (32.7)	232/1,160 (20.0)	579/1,002 (57.8)	428/1,003 (42.7)	660/999 (66.1)
Did not complete primary	655/2,936 (22.3)	1,022/1,992 (51.3)	389/1,999 (19.4)	90/1,373 (6.6)	141/544 (25.9)	445/1,932 (23.1)	354/1,001 (35.4)	510/1,000 (51.0)	153/1,160 (13.2)	114/1,002 (11.4)	234/1,003 (23.3)	195/993 (19.5)
Completed primary	979/2,936 (33.3)	370/1,992 (18.6)	415/1,999 (20.7)	460/1,373 (33.5)	267/544 (49.1)	965/1,932 (50.1)	229/1,001 (22.9)	122/1,000 (12.2)	303/1,160 (26.1)	259/1,002 (25.8)	212/1,003 (21.1)	116/993 (11.6)
Secondary	728/2,936 (24.8)	135/1,992 (6.8)	713/1,999 (35.5)	481/1,373 (35.0)	36/544 (6.6)	266/1,932 (13.8)	99/1,001 (9.9)	25/1,000 (2.5)	335/1,160 (28.9)	45/1,002 (4.5)	87/1,003 (8.7)	26/993 (2.6)
Tertiary	499/2,936 (17.0)	73/1,992 (3.7)	410/1,999 (22.2)	305/1,373 (2.9)	16/544 (4.8)	93/1,932 (9.2)	92/1,001 (9.2)	16/1,000 (1.6)	137/1,160 (11.8)	5/1,002 (0.5)	42/1,003 (4.2)	2/993 (0.2)
Occasionally going hungry through lack of food, *n* (%)	140/2,933 (4.8)	240/1,989 (12.1)	32/1,995 (1.6)	63/1,365 (4.6)	74/547 (13.5)	111/1,862 (6.0)	39/999 (3.9)	85/993 (8.6)	9/1,160 (0.0)	12/1,002 (1.2)	207/995 (20.8)	141/999 (14.1)
Central obesity, *n* (%)	1,063/2,921 (36.4)	907/1,986 (45.7)	871/1,605 (54.3)	602/1,364 (44.1)	214/550 (38.9)	714/1,518 (47.0)	568/992 (57.3)	406/988 (41.1)	530/1,149 (46.1)	158/1,002 (15.8)	174/990 (17.6)	92/956 (9.6)
Smoking status, *n* (%)												
Never smoked	1,612/2,936 (54.9)	1,049/2,009 (52.2)	1,454/2,002 (72.6)	1,119/1,374 (81.4)	482/551 (87.5)	1,061/1,900 (55.8)	648/1,003 (64.6)	729/1,000 (72.9)	875/1,160 (75.4)	666/1,002 (66.5)	730/997 (73.2)	469/999 (46.9)
Ex-smoker	759/2,936 (25.9)	711/2,009 (35.4)	444/2,002 (22.1)	201/1,374 (14.6)	55/551 (10.0)	624/1,900 (32.8)	246/1,003 (24.5)	200/1,000 (20.0)	92/1,160 (7.9)	31/1,002 (3.1)	86/997 (8.6)	42/999 (4.2)
Current smoker	565/2,936 (19.2)	249/2,009 (12.4)	104/2,002 (5.2)	54/1,374 (3.9)	14/551 (2.5)	215/1,900 (11.3)	109/1,003 (10.9)	71/1,000 (7.1)	193/1,160 (16.6)	305/1,002 (30.4)	181/997 (18.2)	488/999 (48.8)
Diabetes—self-reported physician diagnosis, *n* (%)	543/2,928 (18.5)	281/2,007 (14.0)	642/2,002 (32.1)	119/1,371 (8.7)	54/551 (9.8)	309/1,926 (16.0)	246/1,002 (24.6)	189/1,000 (18.9)	195/1,159 (16.8)	9/1,002 (0.9)	121/1,004 (12.1)	66/999 (6.6)
Hypertension—meets WHO/ISH criteria and/or self-reported physician diagnosis, *n* (%)	2,173/2,940 (73.9)	1,537/2,000 (76.9)	1,501/1,880 (79.8)	725/1,378 (52.6)	235/551 (42.6)	1,423/1,789 (79.5)	694/1,003 (69.2)	565/998 (56.6)	734/1,155 (63.5)	570/1,002 (56.9)	687/1,001 (68.6)	445/976 (45.6)
Self-reported stroke, *n* (%)	230/2,935 (7.8)	175/2,005 (8.7)	168/2,001 (8.4)	112/1,373 (8.2)	20/550 (3.6)	135/1,920 (7.0)	67/1,003 (6.7)	74/1,000 (7.4)	109/1,160 (9.4)	18/1,002 (1.8)	20/1,004 (2.0)	11/988 (1.1)
10/66 DRG dementia diagnosis, *n* (%)	316/2,931 (10.7)	235/2,011 (11.7)	233/1,998 (11.6)	129/1,376 (9.4)	36/552 (6.5)	140/1,964 (7.1)	86/1,003 (8.6)	85/1,000 (8.5)	81/1,160 (7.0)	56/1,002 (5.6)	75/1,005 (7.5)	106/999 (10.6)
ICD-10 depressive episode, *n* (%)	144/2,936 (4.9)	278/1,937 (13.8)	47/2,003 (2.3)	87/1,376 (6.3)	16/536 (2.9)	107/1,928 (5.5)	47/998 (4.7)	45/992 (4.5)	3/1,157 (0.3)	7/998 (0.7)	39/986 (3.9)	126/999 (12.6)
>3 physical impairments, *n* (%)	292/2,938 (9.9)	465/2,009 (23.1)	429/2,003 (21.4)	224/1,380 (16.2)	40/550 (7.3)	489/1,932 (24.9)	158/1,003 (15.8)	185/1,000 (18.5)	208/1,160 (17.9)	39/1,002 (3.9)	41/1,004 (4.1)	168/999 (16.8)

Variation in denominators accounted for by participants with missing data.

10/66 DRG, 10/66 Dementia Research Group; ISH, International Society of Hypertension; SD, standard deviation.

### Prevalence of intrinsic capacity

In the pooled dataset, capacity was least likely to be retained with respect to locomotion (neuromusculoskeletal capacity) (71.2%), vision (71.3%), cognition (73.5%), and mood (psychological capacity) (74.1%), and most likely to be retained for continence (96.5%). Just 29.6% retained full capacity across all domains (Table 2). This proportion varied between 25% and 39% in most sites, with low outliers in rural India (11.0%) and the Dominican Republic (15.0%) and a high outlier in urban China (62.8%). There was considerable variation in the prevalence of individual capacities among sites, mostly arising from the China sites; retention of locomotion capacity was exceptionally low in rural China, cognition was well preserved in urban China, and undernutrition and low mood were rarely encountered in either site. Following direct standardisation for age and gender, there was little difference between standardised and crude prevalence, and differences in the demographic composition of the catchment area samples clearly did not account for the variations in prevalence of intrinsic capacities (Tables 1.1 to 1.8 in S2 Appendix).

**Table 2:** Prevalence of individual capacities, and full capacity, by site.

Site	Neuromusculoskeletal capacity (%)	Vitality (nutrition)	Sensory capacity—vision	Sensory capacity—hearing	Cognitive capacity	Psychological capacity	Continence	Full capacity
Cuba, *n* = 2,944	1,713/2,938 (58.6%)	2,580/2,892 (87.8%)	2,063/2,932 (70.4%)	2,626/2,935 (89.5%)	2,287/2,944 (77.7%)	2,210/2,892 (76.4%)	2,801/2,916 (96.1%)	842/2,944 (28.6%)
Dominican Republic, *n* = 2,011	807/1,981 (42.1%)	1,671/1,996 (83.7%)	1,196/2,008 (59.6%)	1,659/2,008 (82.6%)	1,404/2,011 (69.8%)	1,237/1,994 (62.0%)	1,912/2,009 (95.2%)	301/2,009 (15.0%)
Puerto Rico, *n* = 2,009	1,292/1,520 (85.0%)	1,479/1,644 (90.0%)	1,467/2,002 (73.3%)	1,645/2,002 (82.2%)	1,495/2,009 (74.4%)	1,578/1,909 (82.7%)	1,913/2,000 (95.7%)	791/2,009 (39.4%)
Peru (urban), *n* = 1,381	894/1,374 (65.1%)	1,116/1,371 (81.4%)	921/1,376 (66.9%)	1,070/1,376 (77.8%)	1,167/1,381 (84.5%)	945/1,335 (70.8%)	1,317/1,379 (95.5%)	384/1,381 (27.8%)
Peru (rural), *n* = 552	411/550 (74.7%)	444/551 (80.6%)	352/551 (63.9%)	457/551 (82.9%)	436/552 (79.0%)	404/548 (73.7%)	543/550 (98.7%)	143/550 (25.9%)
Venezuela, *n* = 1,965	1,188/1,458 (81.5%)	1,304/1,600 (81.5%)	1,155/1,930 (59.8%)	1,632/1,937 (84.3%)	1,617/1,965 (82.3%)	1,371/1,945 (70.5%)	1,900/1,962 (96.8%)	609/1,969 (31.0%)
Mexico (urban), *n* = 1,003	883/940 (93.9%)	888/999 (88.9%)	716/1,003 (71.4%)	799/1,003 (79.7%)	728/1,003 (72.6%)	684/996 (68.7%)	975 /1,003 (97.2%)	342/1,003 (34.1%)
Mexico (rural), *n* = 1,000	854/913 (93.5%)	806/998 (80.8%)	641/999 (64.2%)	769/1,000 (76.9%)	617/1,000 (61.7%)	731/990 (73.8%)	982/1,000 (98.2%)	252/1,000 (25.2%)
China (urban), *n* = 1,160	1,021/1,160 (88.0%)	1,136/1,154 (98.4%)	962/1,160 (82.9%)	1,013/1,160 (81.3%)	1,048/1,160 (90.3%)	1,082/1,126 (96.1%)	1,099/1,158 (94.9%)	728/1,160 (62.8%)
China (rural), *n* = 1,002	338/1,001 (33.8%)	989/1,002 (98.7%)	937/1,002 (93.5%)	911/1,002 (90.9%)	820/1,002 (81.8%)	959/974 (98.5%)	981/1,002 (97.9%)	262/1,002 (26.1%)
India (urban), *n* = 1,005	906/997 (90.9%)	657/995 (66.0%)	913/1,004 (90.9%)	971/1,003 (96.8%)	574/1,005 (57.1%)	610/1,000 (61.0%)	975/994 (98.1%)	275/1,005 (27.4%)
India (rural), *n* = 999	907/999 (90.8%)	647/979 (66.1%)	776/999 (77.7%)	844/999 (84.5%)	332/999 (33.2%)	532/954 (55.8%)	978/997 (98.1%)	120/999 (12.0%)
Total, *n* = 17,031	11,214/15,751 (71.2%)	13,717/16,227 (84.5%)	12,099/16,966 (71.3%)	14,396/16,976 (84.8%)	12,525/17,031 (73.5%)	12,343/16,663 (74.1%)	16,376/16,970 (96.5%)	5,049/17,031 (29.6%)

Variation in denominators accounted for by participants with missing data.

**Fig 1:**
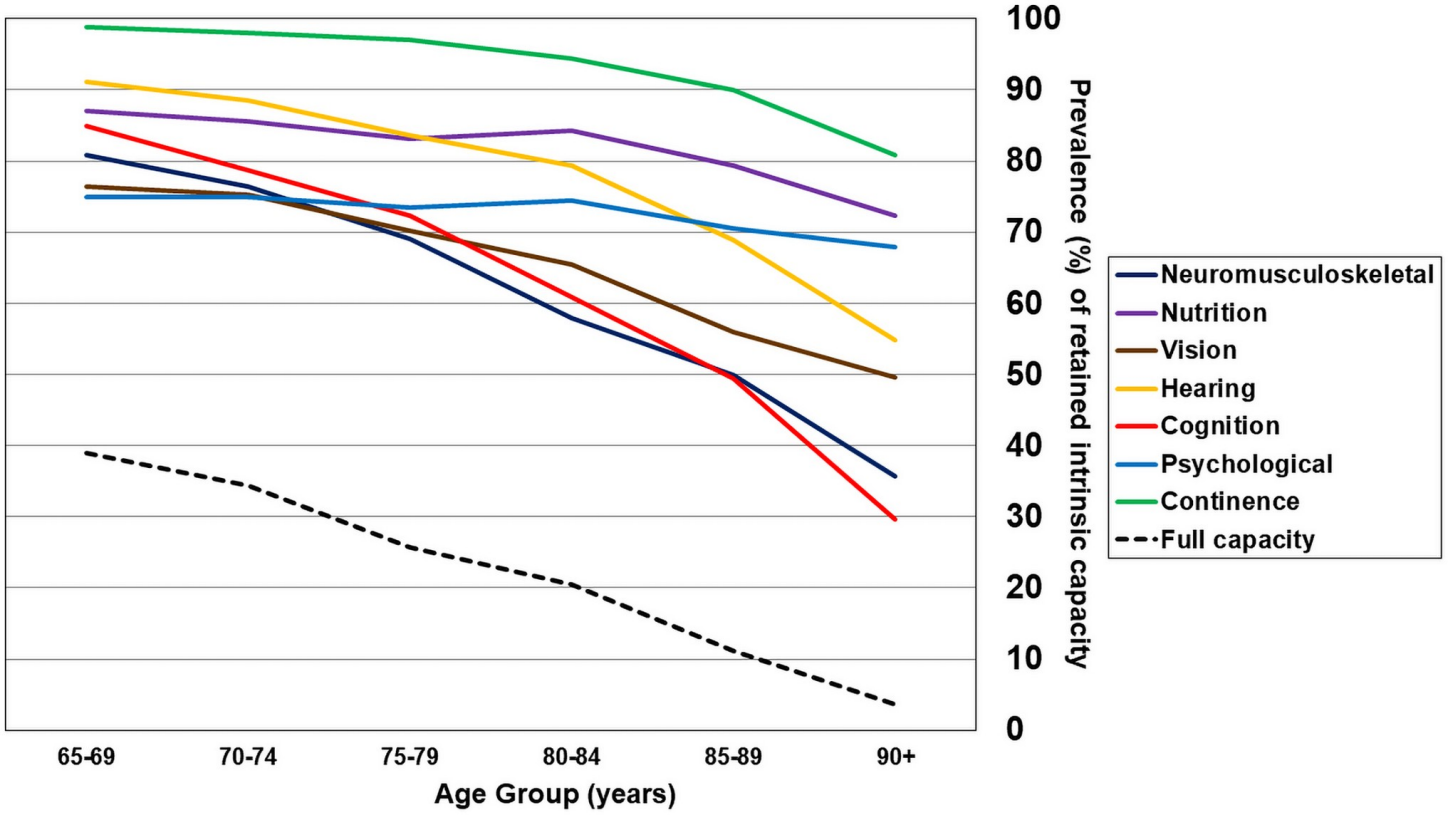
Prevalence (percent) of retained intrinsic capacity, by age group.

**Fig 2:**
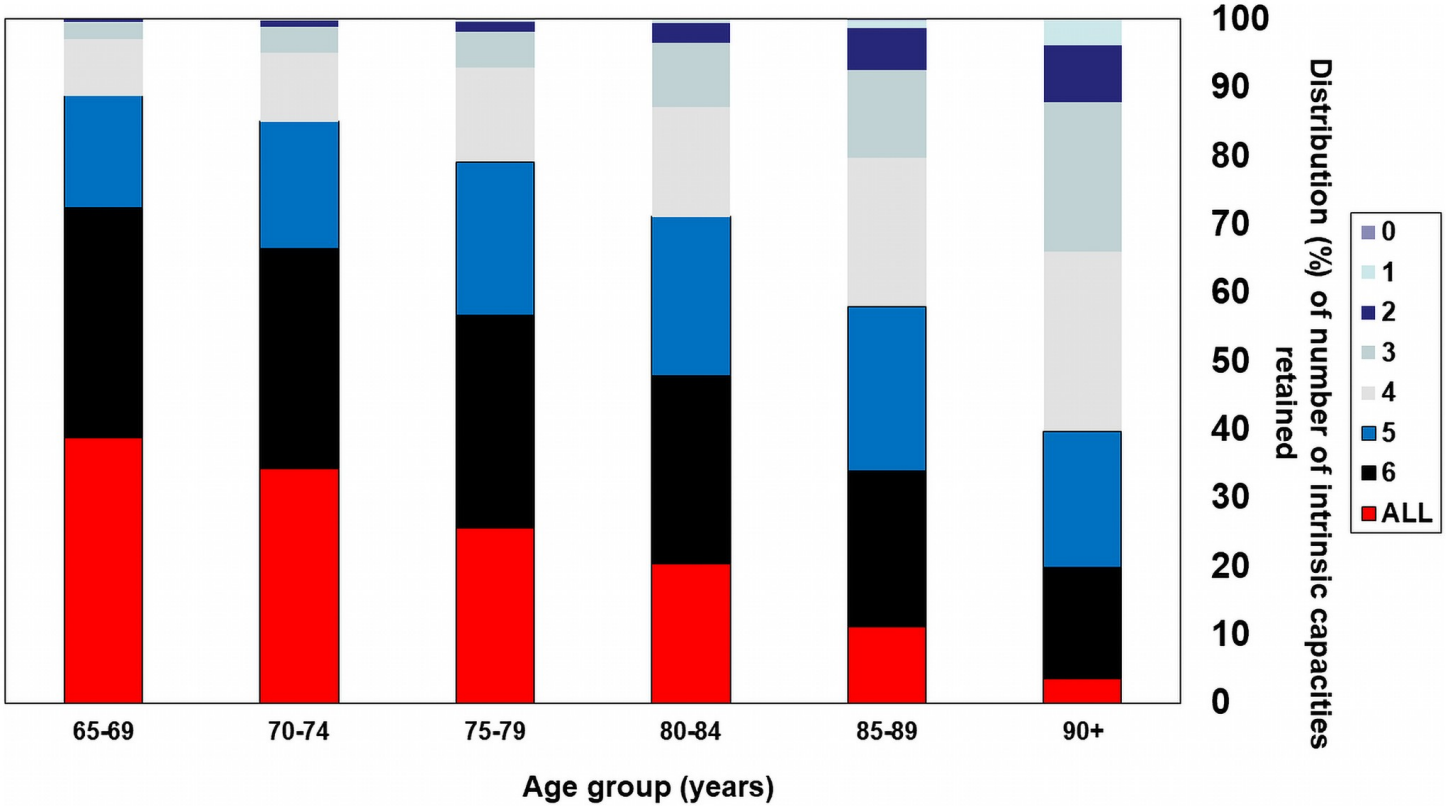
Distribution (percent) of number of intrinsic capacities retained, by age group.

The proportion retaining capacity decreased significantly with age for all individual capacities studied, other than psychological capacity (Fig 1). For continence, hearing, and vision, DICs were more pronounced at age 80 years and over. The proportion retaining full capacity declined linearly with age from 38.9% at age 65–69 years, to 3.6% for those aged 90 years and over. Loss of capacity in multiple domains was also more common in older age groups (Fig 2); loss of capacity in 4 or more domains was detected in 11.3% of those aged 65–69 years, and in 60.2% of those aged 90 years or over.

### Characteristics of those with declines in intrinsic capacity

In principle, all those with DIC affecting 1 or more domains (the target group) might benefit from the ICOPE intervention [4]. Overall, 11,982 older people (70.4%) met this criterion, with the proportion by site varying between 37.2% (urban China) and 88.0% (rural India), with other sites clustering closely around the median of 72.4% (25th centile 67.4%, 75th centile 74.4%). Among those with DIC, 4,541 (37.9%) were neither frail nor in need of care, a further 4,193 (35.0%) were pre-frail, 1,743 (10.2%) were frail, and 1,505 (8.8%) had established needs for care. The characteristics of the target group are shown in Table 3, further broken down by frailty status and dependence (needs for care). Those targeted (with DIC) were on average 3 years older than those not selected, and mean age increased monotonically from the DIC only, to the pre-frail, frail, and dependent groups. Women were over-represented (65.7% among those with DIC versus 54.6% in the group with no DIC). Poverty and disadvantage were particularly marked among those with DIC; those in the DIC target group had nearly 3 times the prevalence of food insecurity (8.5% versus 2.9%), and most had not completed primary education (50.9% versus 29.8%). Morbidity and disability were also concentrated among those in the target group; this was very marked for depression (7.9% versus 0.1%), dementia (12.5% versus 0.4%), and stroke (7.9% versus 3.4%), and these conditions were further concentrated among those who were also frail or had needs for care. Diabetes and health service utilisation and costs were moderately associated with DIC. Other than physical activity and diet, lifestyle risk factors for chronic disease were not concentrated among those with DIC.

**Table 3:** Characteristics of the target group with DIC, further stratified by frailty and dependence (needs for care).

Characteristic	No DIC, *n* = 5,049	DIC (target group), *n* = 11,982	DIC with no frailty or needs for care, *n* = 4,541	DIC and pre-frail, *n* = 4,193	DIC and frail, *n* = 1,743	DIC and needing care, *n* = 1,505	DIC versus no DIC: Chi-squared or *F* statistic (1 degree of freedom)	Test for trend across categories: Chi-squared or *F* statistic (1 degree of freedom)
Age in years, mean (SD)	72.0 (5.6)	75.1 (7.3)	73.7 (6.5)	74.3 (7.0)	75.9 (7.2)	80.1 (8.0)	691.7, *p <* 0.001^1^	464.2, *p <* 0.001^1^
Male gender	2,285 (45.4%)	4,063 (34.3%)	1,654 (37.7%)	1,366 (32.6%)	568 (32.6%)	475 (31.6%)	183.3, *p <* 0.001^2^	176.8, *p <* 0.001^2^
Did not complete primary education	1,495 (29.8%)	5,997 (50.9%)	2,274 (51.9%)	2,060 (49.2%)	866 (49.9%)	797 (53.7%)	636.4, *p <* 0.001^2^	358.1, *p <* 0.001^2^
Food insecurity	146 (2.9%)	991 (8.5%)	235 (5.4%)	400 (9.6%)	231 (13.4%)	125 (8.4%)	168.6, *p <* 0.001^2^	223.4, *p <* 0.001^2^
ICD-10 depression	3 (0.1%)	934 (7.9%)	96 (2.2%)	324 (7.7%)	305 (17.5%)	209 (13.9%)	413.3, *p <* 0.001^2^	961.3, *p <* 0.001^2^
Dementia	21 (0.4%)	1,483 (12.5%)	252 (5.7%)	293 (7.0%)	190 (10.9%)	748 (49.8%)	638.0, *p <* 0.001^2^	2,350.2, *p <* 0.001^2^
Stroke	172 (3.4%)	929 (7.9%)	188 (4.3%)	259 (6.2%)	146 (8.4%)	336 (22.5%)	113.2, *p <* 0.001^2^	521.7, *p <* 0.001^2^
Chronic obstructive pulmonary disease	148 (2.9%)	661 (5.6%)	176 (4.0%)	244 (5.8%)	134 (7.7%)	107 (7.2%)	53.7, *p <* 0.001^2^	96.8, *p <* 0.001^2^
Diabetes	743 (14.8%)	1,999 (16.9%)	550 (12.5%)	752 (18.0%)	356 (20.5%)	341 (22.9%)	11.9, *p <* 0.001^2^	94.2, *p <* 0.001^2^
Hypertension	3,262 (66.2%)	7,944 (68.4%)	2,887 (66.9%)	2,800 (67.9%)	1,224 (70.9%)	1,033 (70.9%)	7.0, *p* = 0.008^2^	19.1, *p <* 0.001^2^
Uncontrolled hypertension	2,025 (41.1%)	4,928 (43.7%)	1,969 (46.7%)	1,646 (41.3%)	714 (42.5%)	599 (42.8%)	1.5, *p* = 0.21^2^	1.7, *p* = 0.19^2^
Central obesity	1,970 (41.5%)	4,304 (38.5%)	1,434 (34.0%)	1,632 (41.1%)	705 (42.3%)	533 (39.7%)	12.9, *p <* 0.001^2^	1.1, *p* = 0.30^2^
Low physical activity	889 (17.7%)	4,795 (40.7%)	1,183 (27.0%)	1,515 (36.3%)	926 (53.3%)	1,171 (78.6%)	831.0, *p <* 0.001^2^	2,167.3, *p <* 0.001^2^
Less than daily fruit and vegetable consumption	891 (17.8%)	2,974 (25.4%)	893 (20.5%)	1,172 (28.2%)	516 (30.0%)	393 (26.6%)	112.6, *p <* 0.001^2^	160.5, *p <* 0.001^2^
Hazardous alcohol use	131 (2.8%)	344 (3.1%)	142 (3.4%)	109 (2.8%)	53 (3.3%)	40 (2.8%)	0.96, *p* = 0.33^2^	0.02, *p* = 0.88^2^
Current smoker	738 (14.7%)	1,801 (15.3%)	823 (18.8%)	569 (13.6%)	252 (14.5%)	157 (10.5%)	6.3, *p* = 0.04^2^	86.2, *p <* 0.001^2^
Used healthcare services in last 3 months	2,348 (46.6%)	6,573 (55.6%)	2,176 (49.6%)	2,437 (58.1%)	1,084 (62.2%)	876 (58.2%)	112.2, *p <* 0.001^2^	181.1, *p <* 0.001^2^
Out-of-pocket healthcare cost in top 10th by site	414 (8.2%)	1,295 (11.0%)	350 (8.0%)	446 (10.6%)	222 (12.7%)	277 (18.5%)	28.4, *p <* 0.001^2^	140.2, *p <* 0.001^2^
Total healthcare costs in top 10th by site	281 (6.6%)	1,172 (11.0%)	338 (8.4%)	407 (10.8%)	202 (13.0%)	225 (17.3%)	65.6, *p <* 0.001^2^	158.3, *p <* 0.001^2^
Pain scale, mean (SD)	2.7 (3.1)	4.2 (4.0)	3.3 (3.4)	4.5 (4.0)	5.5 (4.2)	4.1 (4.5)	537.5, *p <* 0.001^1^	261.6, *p <* 0.001^1^
WHODAS 2.0 disability, mean (SD)	3.8 (7.9)	17.0 (20.8)	8.5 (12.0)	13.0 (14.6)	21.3 (18.9)	48.0 (13.0)	1,802.3, *p <* 0.001^1^	2,848.1, *p <* 0.001^1^

Values are *n* (percent) unless otherwise noted.

^1^ANOVA test (*F* statistic) comparing 2 means (penultimate column) or for trend in means across groups (last column).

^2^Chi-squared test comparing 2 proportions (penultimate column) or for trend in proportions across groups (last column).

DIC, decline in intrinsic capacity; ICD -0, International Classification of Diseases–10thRevision; SD, standard deviation.

### Predictors of incident dependence

In total, 12,939 participants who had no needs for care at baseline were included in the dependence cohort, of whom 9,053 (70.0%) were re-interviewed, and 1,684 (13.0%) had died. The 2,202 participants (17.0%) who were lost to follow-up (i.e., not re-interviewed, but not known to have died) were excluded from the analysis. Of these, 726 had refused, 591 could not be traced, 578 were traced but could not be contacted for re-interview (‘uncontactable’), and 307 did not have a reason for LTFU recorded. Sufficient information was available from the verbal autopsy interview to predict needs for care before death for 1,328 of the 1,684 who had died (78.9%). An outcome of dependence-free death (competing risk) was allocated to 832 deceased, and onset of dependence before death to 496. A further 1,400 incident cases were identified among those re-interviewed. Therefore, 1,896 incident cases were identified in 38,320 person-years of follow-up, a rate of 49.5 per 1,000 person-years. We have provided further information on cohort attrition, including a cohort flow diagram (Fig 2.1 in S2 Appendix) and information on attrition by site (Table 2.4 in S2 Appendix).

We also carried out additional analyses suggested by reviewers to explore the potential for attrition bias (S2 Appendix). In summary, in a pooled analysis comparing those who were re-interviewed, those who were lost to follow-up, and those who had died, those who had died were older, had poorer cognitive performance, and were more likely to be male, to be less educated, to be frail, to have experienced significant disability, and to have more DICs at baseline (Table 2.5 in S2 Appendix). Those who were lost to follow-up were more akin to those who were re-interviewed in all these respects. Nevertheless, LTFU arguably posed the greatest risk for bias, since dependence outcomes were recorded from verbal autopsy interview data for most of those who had died; LTFU accounted for 84.3% (2,202/2,613) of cohort members who could not be included in the analysis (Table 2.4 in S2 Appendix). Accordingly, in a logistic regression analysis, we compared directly those who were re-interviewed with those lost to follow-up, mutually controlling for potential determinants of LTFU (age, gender, education, and number of domains affected by DIC—which might also be among the determinants of incident dependence). The only consistent independent association across sites was between older age and LTFU (pooled adjusted odds ratio [aOR] 1.07, 95% CI 1.02–1.12, per 5-year increment in age) (Table 2.6 in S2 Appendix). Number of DICs was positively associated with LTFU in Puerto Rico, and inversely associated in rural Mexico, but with no overall effect across sites (pooled aOR 0.98, 95% CI 0.94–1.03). There was also no evidence that frailty predicted LTFU, when we substituted this exposure for number of DICs in the model (pooled aOR 1.06, 95% CI 0.91–1.23).

Mutually controlling for all other domains of intrinsic capacity, and further adjusting for age, gender, and education, declines in cognitive capacity (pooled aSHR 2.56, 95% CI 2.26–2.90) and continence (pooled aSHR 3.32, 95% CI 2.48–4.44) were strongly associated with incident dependence (Table 4). Declines in psychological capacity (pooled aSHR 1.33, 95% CI 1.18–1.51), locomotor capacity (pooled aSHR 1.42, 95% CI 1.26–1.60), and nutrition (pooled aSHR 1.21, 95% CI 1.04–1.41) were moderately associated. Neither of the 2 sensory capacities (vision and hearing) was associated with incident dependence.

**Table 4:** Adjusted associations of individual declines in intrinsic capacity with incident dependence.

Site or measure	Cognitive capacity	Psychological capacity	Continence	Locomotion	Hearing	Vision	Nutrition
Cuba	2.93 (2.14–4.01)	1.40 (1.03–1.88)	1.81 (0.58–5.63)	1.46 (1.11–1.91)	0.99 (0.68–1.46)	0.93 (0.69–1.23)	1.39 (0.99–1.95)
Dominican Republic	2.51 (1.95–3.23)	1.14 (0.89–1.45)	3.63 (1.86–7.10)	1.25 (0.99–1.58)	0.99 (0.76–1.29)	1.07 (0.85–1.33)	1.33 (1.00–1.76)
Puerto Rico	3.23 (2.30–4.55)	1.43 (0.98–2.08)	1.93 (1.14–3.29)	1.68 (1.20–2.36)	0.96 (0.66–1.41)	1.07 (0.77–1.24)	0.93 (0.55–1.59)
Peru urban	4.03 (2.47–6.55)	1.20 (0.80–1.81)	9.07 (4.82–17.09)	1.16 (0.79–1.72)	0.88 (0.55–1.41)	1.14 (0.76–1.71)	1.00 (0.61–1.65)
Peru rural	3.93 (1.69–9.13)	0.60 (0.27–1.32)	DNC^1^	1.33 (0.69–2.56)	1.13 (0.50–2.56)	1.55 (0.81–2.98)	1.53 (0.72–3.24)
Venezuela	2.52 (1.76–3.61)	1.47 (1.02–2.10)	2.93 (1.36–6.32)	1.93 (1.37–2.72)	1.32 (0.87–2.02)	1.18 (0.85–1.63)	1.16 (0.79–1.72)
Mexico urban	1.91 (1.15–3.16)	1.43 (0.93–2.20)	4.42 (1.39–14.05)	1.61 (0.80–3.22)	0.69 (0.40–1.20)	1.34 (0.87–2.09)	0.82 (0.37–1.82)
Mexico rural	2.33 (1.46–3.74)	1.55 (0.98–2.47)	DNC^1^	1.89 (1.03–3.46)	0.97 (0.59–1.58)	1.00 (0.66–1.52)	1.16 (0.69–1.96)
China urban	0.76 (0.35–1.65)	1.71 (0.67–4.36)	1.50 (0.31–7.35)	1.65 (0.97–2.80)	1.41 (0.95–2.09)	1.28 (0.89–1.84)	DNC^1^
China rural	1.98 (1.36–2.88)	3.94 (1.70–9.13)	DNC^1^	1.05 (0.73–1.51)	0.99 (0.58–1.71)	1.20 (0.65–2.21)	DNC^1^
Pooled effect size	2.56 (2.26–2.90)	1.33 (1.18–1.51)	3.32 (2.48–4.44)	1.42 (1.26–1.60)	1.03 (0.90–1.17)	1.11 (0.99–1.24)	1.21 (1.04–1.41)
Heterogeneity—Higgins *I*^2^ percent (95% CI)	54 (6–77)	33 (0–68)	63 (16–84)	12 (0–52)	0 (0–62)	0 (0–62)	0 (0–68)

From a competing risks Cox proportional hazards regression, controlling for age, gender, education, and all other declines in intrinsic capacity, generating adjusted subhazard ratios and their 95% confidence intervals.

^1^Model did not converge (DNC) (low exposure prevalence).

DICs affecting 1 or more domains were associated with incident dependence (pooled aSHR 1.91, 95% CI 1.69–2.17) (Table 5). Although the increased risk was somewhat concentrated among those who were frail (versus no DIC, pooled aSHR 2.90, 95% CI 2.44–3.45), it was still apparent among the larger sub-groups with DIC who were neither pre-frail nor frail (versus no DIC, pooled aSHR 1.72, 95% CI 1.50–2.17) and who were considered pre-frail (versus no DIC, pooled aSHR 1.89, 95% CI 1.64–2.19).

**Table 5:** The adjusted associations of declines in intrinsic capacity (DICs), and DICs stratified by frailty status, with incident dependence.

Site or measure	No DIC, *n* = 5,049	DIC, *n* = 11,982	DIC, with no frailty or needs for care, *n* = 4,541	DIC and pre-frail, *n* = 4,193	DIC and frail, *n* = 1,743
Cuba	1 (ref)	2.71 (1.89–3.91)	2.36 (1.59–3.50)	2.75 (1.84–4.13)	3.61 (2.35–5.56)
Dominican Republic	1 (ref)	1.51 (1.08–2.13)	1.37 (0.95–1.98)	1.49 (1.04–2.15)	1.88 (1.26–2.78)
Puerto Rico	1 (ref)	2.21 (1.67–2.94)	2.13 (1.50–3.02)	2.08 (1.49–2.90)	2.68 (1.83–3.94)
Peru urban	1 (ref)	3.05 (1.42–6.52)	2.83 (1.26–6.38)	2.80 (1.25–6.29)	3.68 (1.63–8.31)
Peru rural	1 (ref)	2.91 (0.99–8.56)	2.91 (0.91–9.29)	1.91 (0.60–6.06)	6.22 (1.90–20.44)
Venezuela	1 (ref)	2.27 (1.59–3.23)	1.82 (1.21–2.74)	2.25 (1.53–3.32)	3.86 (2.46–6.04)
Mexico urban	1 (ref)	1.89 (1.17–3.06)	1.51 (0.82–2.80)	1.60 (0.94–2.73)	3.61 (1.98–6.59)
Mexico rural	1 (ref)	1.84 (1.02–3.30)	1.46 (0.76–2.81)	1.55 (0.81–2.98)	3.40 (1.77–6.56)
China urban	1 (ref)	1.56 (1.17–2.09)	1.54 (1.11–2.14)	1.76 (1.17–2.64)	1.12 (0.38–3.33)
China rural	1 (ref)	1.30 (0.87–1.95)	1.27 (0.84–1.93)	1.26 (0.77–2.08)	2.37 (1.83–3.94)
Pooled effect size	1 (ref)	1.91 (1.69–2.17)	1.72 (1.50–1.98)	1.89 (1.64–2.19)	2.90 (2.44–3.45)
Heterogeneity—Higgins *I*^2^ percent (95% CI)		39 (0–71)	15 (0–56)	12 (0–54)	32 (0–68)

From a competing risks Cox proportional hazards regression, controlling for age, gender, and education, generating adjusted subhazard ratios and their 95% confidence intervals.

When comparing the crude and mutually adjusted effects of frailty (versus no frailty) and number of DICs (per domain affected), number of DICs was the more independent predictor of incident dependence. The effect of frailty was considerably attenuated after controlling for number of DICs (from pooled aSHR 1.78 to aSHR 1.21), whereas that of number of DICs was only moderately reduced when controlling for frailty (from pooled aSHR 1.35 per DIC to aSHR 1.32) (Table 6).

**Table 6:** The adjusted associations of frailty and declines of intrinsic capacity (number of domains affected, per domain) with incident dependence.

Site or measure	Frailty^1^	Number of capacities affected, per domain^1^	Frailty^2^	Number of capacities affected, per domain^3^
Cuba	1.69 (1.25–2.28)	1.39 (1.27–1.53)	1.07 (0.74–1.54)	1.38 (1.23–1.55)
Dominican Republic	1.38 (1.07–1.77)	1.27 (1.17–1.37)	0.95 (0.72–1.26)	1.28 (1.17–1.39)
Puerto Rico	1.67 (1.21–2.31)	1.42 (1.28–1.56)	1.01 (0.68–1.49)	1.41 (1.25–1.59)
Peru urban	1.53 (1.01–2.31)	1.28 (1.13 (1.47)	1.03 (0.63–1.71)	1.28 (1.09–1.50)
Peru rural	3.18 (1.70–5.97)	1.35 (1.08–1.68)	2.42 (1.12–5.21)	1.20 (0.94–1.53)
Venezuela	2.26 (1.59–3.20)	1.47 (1.33–1.63)	1.34 (0.90–2.01)	1.42 (1.26–1.60)
Mexico urban	2.33 (1.44–3.74)	1.26 (1.07–1.50)	1.98 (1.18–3.32)	1.16 (0.97–1.39)
Mexico rural	2.45 (1.62–3.70)	1.35 (1.17–1.55)	1.90 (1.20–3.00)	1.25 (1.06–1.46)
China urban	1.04 (0.35–3.07)	1.34 (1.09–1.64)	0.94 (0.34–2.60)	1.34 (1.09–1.64)
China rural	1.81 (0.94–3.50)	1.28 (1.06–1.54)	1.45 (0.71–2.94)	1.24 (1.03–1.50)
Pooled effect size	1.78 (1.57–2.01)	1.35 (1.33–1.43)	1.21 (1.05–1.40)	1.32 (1.26–1.37)
Heterogeneity—Higgins *I*^2^ percent (95% CI)	37 (0–70)	0 (0–62)	42 (0–72)	0 (0–62)

From competing risks Cox proportional hazards regressions, generating adjusted subhazard ratios and their 95% confidence intervals.

^1^Controlling for age, gender, and education.

^2^Controlling for age, gender, education, and number of capacities affected.

^3^Controlling for age, gender, education, and frailty.

### Predictors of mortality

In total, 15,901 participants were included in the mortality cohort, among whom vital status at follow-up was ascertained for 13,936 (87.5%); 2,602 deaths were recorded during 53,911 person-years of follow-up, a mortality rate of 48.2 per 1,000 person-years. We provide further information on cohort attrition (S2 Appendix), including attrition by site (Table 2.1 in S2 Appendix). At the request of reviewers, we also carried out additional analyses to explore the potential for attrition bias. We did not find any evidence, overall, that relevant baseline covariates were associated with failure to ascertain vital status at follow-up for age (pooled aOR 1.02, 95% CI 0.97–1.07), gender (pooled aOR 0.96, 95% CI 0.88–1.06), education (pooled aOR 1.01, 95% CI 0.96–1.07), number of DICs (pooled aOR 0.99, 95% CI 0.95–1.04), frailty (pooled aOR 0.99, 95% CI 0.87–1.04), or dependence (pooled aOR 1.00, 95% CI 0.84–1.20) (Table 2.2 in S2 Appendix). However, there was moderate to high heterogeneity of effect among sites for the health covariates, and evidence that number of DICs and frailty might have been inversely associated with attrition in the Dominican Republic, and positively associated with attrition in Puerto Rico. Replicating an analysis reported previously for a different but overlapping set of covariates [40], we found that the predicted probability of mortality was lower among those for whom vital status was not ascertained than among those for whom it was ascertained in urban China (0.181 versus 0.226, *p* = 0.002) and in the Dominican Republic (0.246 versus 0.271, *p* = 0.02), but similar between the 2 groups in other sites (Table 2.3 in S2 Appendix).

**Fig 3:**
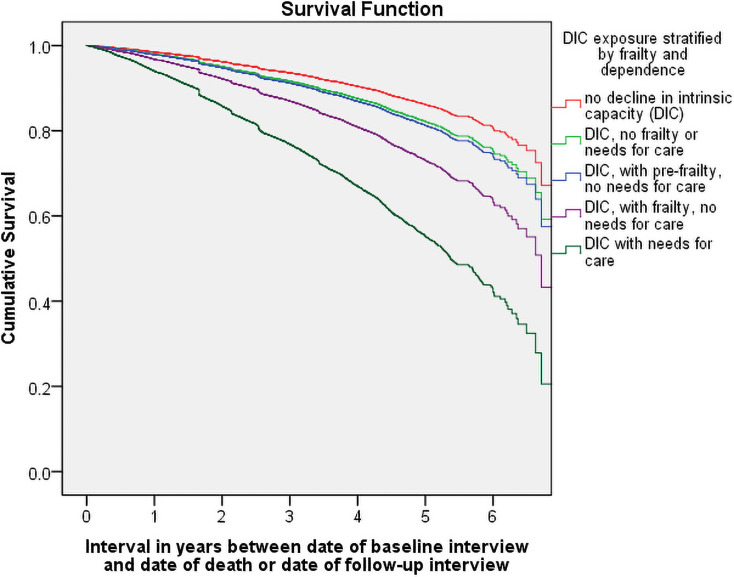
Cumulative survival probability by intrinsic capacity, frailty, and need for care.

Cumulative survival worsened progressively across sub-groups from those with no DIC to those who had DIC only, had DIC and were also pre-frail, were frail, and were dependent (Fig 3). Detailed site-specific and meta-analytically pooled results from modelling of predictors of mortality are provided in S1 Table. In summary, having controlled for age, gender, and education, mortality was strongly influenced by DIC (any DIC versus no DIC), with negligible heterogeneity across sites (pooled aHR 1.66, 95% CI 1.49–1.85; *I*^2^ = 0, 95% CI 0–60). Mortality risk was considerably elevated in the frail (versus no DIC, pooled aHR 2.20, 95% CI 1.89–2.56; *I*^2^ = 0, 95% CI 0–60) and dependent (versus no DIC, pooled aHR 3.92, 95% CI 3.43–4.49; *I*^2^ = 33, 95% CI 0–67) sub-groups, with only around half surviving to 6 years (Fig 3). However, there was also a small increase in mortality in those with DIC who were neither pre-frail nor frail (versus no DIC, pooled aHR 1.27, 95% CI 1.12–1.45; *I*^2^ = 39, 95% CI 0–70) and in those who were pre-frail (versus no DIC, pooled aHR 1.42, 95% CI 1.24–1.63; *I*^2^ = 1, 95% CI 0–61). Although mortality risk increased monotonically with each additional DIC (per domain affected, pooled aHR 1.32, 95% CI 1.28–1.36; *I*^2^ = 44, 95% CI 0–72), this effect was substantially attenuated after controlling for frailty and dependence (pooled aHR 1.16, 95% CI 1.12–1.20; *I*^2^ = 58, 95% CI 17–78). The proportional hazards test suggested some departures from proportional hazards assumptions in some sites. These all related to control covariates (age, or age and gender), and not to the DIC variables. Interactions with time were introduced for those covariates for the sites where this was an issue, and the effects on the pooled estimates were negligible.

## Discussion

In this retrospective analysis of data previously acquired from a prospective population-based cohort study, we showed that, typically, two-thirds to three-quarters of those aged 65 years and over have experienced declines affecting 1 or more domains of intrinsic capacity. DICs were more prevalent in older age groups, as were DICs affecting multiple domains. While DIC was strongly associated with disability, only around one-fifth of those affected were frail or dependent. There was a strong concentration of cases of dementia, stroke, and clinically relevant depression among those with DIC, particularly among those who were already frail or needed care. Hypertension, diabetes, and behavioural risk factors for chronic disease were less strongly associated with intrinsic capacity, with a substantial prevalence among those who were yet to have experienced DIC. DIC (both any decline and number of domains affected) strongly and independently predicted incident dependence and death. The increased risks of dependence were concentrated among those who were frail but, nevertheless, were also substantially higher for the much larger sub-groups yet to become frail. Mortality was particularly high in the frail and dependent groups.

The theoretical conceptualisation of intrinsic capacity as a core component of healthy ageing is a recent development [1]. We believe that our research is among the first to report on the epidemiology of intrinsic capacity in older populations in LMICs, using assessments and indicators that might be applicable to Demographic and Health Surveys and routine care. Our findings of important independent associations between DICs and incident dependence are consistent with those from a causal modelling analysis using data from the English Longitudinal Study of Ageing; in that study intrinsic capacity had a direct relationship with incident loss of activities of daily living, mediating some of the effects of age, gender, wealth, education, and multimorbidity on this outcome. [42] The main strengths of our study include the large population-based samples with a high proportion of responders in most sites and modest missing data. The diverse countries and settings, across 3 continents, allowed us to study and explore variation in the context of a common study protocol, with detailed assessment of physical, mental, and cognitive morbidities, healthcare utilisation, disability, frailty, and needs for care.

There are several limitations. The WHO has yet to finalise the criteria that it will recommend for screening for intrinsic capacity. Both the approach and the thresholds selected are likely to have important impacts on the proportions that screen positive. We would acknowledge some deficiencies in the measures that we had available from our survey, which was not designed with this purpose in mind. Reliance on informant reports of incontinence may have selectively identified more severe cases, hence, perhaps, the low prevalence and the strong association with both prevalent and incident dependence in our study. Brief self-report measures such as the International Consultation on Incontinence Questionnaire (ICIQ) could provide information on aetiology and impact as well as a more realistic sense of population prevalence [43]. Vision and hearing would be better assessed through objective tests of functioning, rather than the self-report and interviewer judgment that we had available. As previously reported, our frailty indicators were operationalised in slightly different ways [30] from those originally suggested [29], and consequent measurement error may have compromised their ability to predict the onset of needs for care. Our outcome measure of care dependence was not formally validated before its introduction in the baseline wave of our surveys. While criterion validation is not feasible, we have accumulated evidence to support construct validity (concurrent and predictive) across the sites where we have been working (S1 Working Paper). The lack of information on inter-rater reliability of the assessment procedure is a further limitation. Finally, we are not able confidently to exclude the possibility that selective attrition may have biased estimates of associations with incident dependence (where 17% of the cohort were lost to follow-up) and mortality (where the outcome could not be ascertained for 12% of the cohort). While, overall, attrition seemed to be random with respect to relevant sociodemographic and health exposures including DIC, there was evidence that this may not have been the case in some sites, where local factors interacting with health exposures may have played a part in determining attrition.

At baseline, we identified those with DIC, and further subdivided these individuals into 4 groups reflecting the common concept of the ‘disability cascade’ through which, with the accumulation and progression of DIC, individuals become frail, experience limitation in core activities of daily living, and require care. The findings from our analysis broadly support the WHO’s strategy to focus upon optimising intrinsic capacity in pursuit of healthy ageing. If the ultimate public health aim is to reduce the future toll of disability and dependence, then most incident cases will arise among those with DIC who are yet to become frail, and a simple count of the domains of intrinsic capacity affected is a better predictor than frailty of the onset of needs for care across the spectrum. Our research confirms the importance of cognitive and mental health in the maintenance of functional independence, and the inclusion of cognitive and psychological capacity in the intrinsic capacity framework ensures that attention is given to brain and mind, alongside physical frailty. Nevertheless, the aim to optimise intrinsic capacity broadens the potential scope of the intervention from the one-fifth of the older population who are already frail or dependent (the focus of most programmes to date) to encompass up to three-quarters of the general population of older people. This raises important questions of appropriate targeting, from perspectives of feasibility and cost-effectiveness.

A comprehensive approach to healthy ageing should involve promotion (optimising health behaviours and structural conditions in society), prevention (managing underlying risk factors and conditions), treatment (aiming at health improvements), rehabilitation (aiming at improvements in functioning, and mitigation of the effects of disability), and palliation (aiming at improvements in quality of life). The salience of each of these activities varies across the disability cascade, with promotion and prevention dominating for those yet to show DIC, treatment and rehabilitation for those with early isolated declines, and rehabilitation and palliation for those who are frail and dependent, some of whom will be nearing the end of life [1]. There will be much blurring across these boundaries. As our data indicate, promotion and prevention are relevant throughout, yet not well covered in ICOPE; integration with the WHO Package of Essential Noncommunicable Disease Interventions (PEN) will be important since cardiovascular risk is high, and detection and control of hypertension and diabetes sub-optimal [35,44,45].

Community healthcare workers (CHWs) have an essential part to play if universal health coverage is to extend to older adults in low-resource settings. There has been interest in extending the roles of this cadre from maternal and child health, sanitation, and infection control to include prevention and control of noncommunicable disease. There is early evidence of feasibility and effectiveness in cardiovascular risk reduction, detecting hypertension and diabetes [46,47], and supporting the management of hypertension [48,49]. Coordinating the integrated care of older people in the community would be a logical next step, capitalising on the CHWs’ unique outreach capacity and knowledge of older people and their families [50]. Assessing intrinsic capacity in older adults does not require specialist skills, and can be done by CHWs in low-resource settings using a structured tool after brief training [50]. Outreach may be necessary for early identification of DICs; in the current analysis, although those with DICs are slightly more likely to use healthcare services, this is mainly confined to those who are also frail or dependent. Outreach will also help to engage those with DICs who are frail or dependent, who may otherwise find it difficult to access services. In an earlier report from the same cohort we highlighted the inequity in access linked to poverty and lack of health insurance [51]. Nevertheless, the challenges of delivering integrated care at scale would be considerable. A fully fledged system might comprise universal screening, followed by more detailed assessment for the two-thirds or more of the older population who could benefit from 1 or more ICOPE interventions. Optimal intervals of reassessment for screen-negative and screen-positive groups would need to be established. This would require a significant diversion of limited healthcare resources, and the cost-effectiveness of the investment is uncertain. Screening would not be warranted if resources and systems were not in place to provide the recommended interventions.

The evidence used to support most of the ICOPE recommendations is indirect [3]. Interventions were mostly evaluated in HICs using experienced senior nurses, physical or occupational therapists, or nutritionists, often with specialist support and full access to secondary care referral pathways. In LMICs, with few specialists, task-shifting to trained and supported non-specialist health workers will be a key feature of any accessible healthcare system for older people. This need not imply an attenuation of effect; in the previously cited systematic review, effect sizes were larger for earlier studies (pre-1993), before systematic approaches to increasing coverage of treatment and care for older people were generalised in HICs [18]. Task-shifting has been shown to be an effective health system innovation for widening access to many different aspects of healthcare in low-resourced settings [52].

Training will be required to ensure rigour and accuracy of screening assessments, distinguishing where possible longer-term decline from acute and/or self-limiting conditions. While many of the ICOPE interventions can be delivered at home by CHWs, there will need to be clear and well-functioning referral pathways for further clinical assessment and treatment of acute and severe cases (‘red flags’), and for provision of optometry and cataract surgery and hearing aid services. Pilot intervention development will be needed for each of the recommendations to confirm feasibility and optimise modes of delivery in different contexts. ICOPE is a complex intervention comprising a package of interventions delivered flexibly in an individualised and person-centred care plan, involving different cadres and different levels of the health system, targeting older people and their carers through direct physical intervention and behavioural and environmental change. Health systems research should focus on the planning, financing, resourcing, and organisational change management that might be required to implement the ICOPE programme at scale. Implementation science will be needed to understand the nature of the potential benefits, the underlying mechanisms, and how these can best be achieved. Ultimately, cluster randomised controlled trials generating evidence on cost-effectiveness are most likely to inform funding decisions. Most importantly, no opportunity should be lost to evaluate pilot projects, and to ‘learn by doing’.

If the WHO’s triple billion targets are to be achieved with equity, then it is essential that focused attention is given to closing the health access and outcome gaps experienced by older people, particularly those disadvantaged by disability, living in poverty, and living in low-resourced areas. Implementation and scale up of the ICOPE programme offers a conceptually simple, bottom-up, and public-health-orientated approach to ensuring that the benefits of universal health coverage and the enjoyment of better health and well-being are fairly distributed and inclusive. Effective implementation will require political will, prioritisation, investment, and health system strengthening and restructuring. Research has a crucial role to play in guiding and evaluating initial attempts at implementation, and in providing the evidence that governments and policymakers require to prioritise action and mobilise investment.

## Supporting information

S1 Appendix 10/66 Dementia Research Group population-based cohort study incidence data analysis plan. (PDF)

S2 Appendix Additional analyses suggested by reviewers. (PDF)

S1 Checklist Strengthening the Reporting of Observational Studies in Epidemiology (STROBE) guideline checklist. (PDF)

S1 Table Adjusted associations of declines in intrinsic capacity (DICs), and DICs stratified by frailty status, with mortality, by site. (PDF)

S1 Working Paper Dependence (needs for care) in the 10/66 Dementia Research Group studies. (PDF)

## Abbreviations

10/66 DRG: 10/66 Dementia Research Group
aHR: adjusted hazard ratio
aOR: adjusted odds ratio
aSHR: adjusted subhazard ratio
CHW: community healthcare worker
CSI-D: Community Screening Instrument for Dementia
DIC: decline in intrinsic capacity
HIC: high-income country
ICOPE: Integrated Care for Older People
LMICs: low- and middle-income countries
LTFU: loss to follow-up
WHO: World Health Organization
