# Supplementary material for: Intrinsic capacity and its associations with incident dependence and mortality in 10/66 Dementia Research Group studies in Latin America, India, and China: A population-based cohort study
Source: PLoS Med. 2021 Sep 14;18(9):e1003097. doi: 10.1371/journal.pmed.1003097 (PMC8439485; doi:10.1371/journal.pmed.1003097)
Supplement: S1 Appendix — (PDF) [file pmed.1003097.s001.pdf]

S1 APPENDIX

10/66 DEMENTIA RESEARCH GROUP POPULATION-BASED COHORT STUDY INCIDENCE DATA ANALYSIS PLAN

Pre-planned outcomes for the 10/66 Dementia Research Group population-based cohort study included mortality, dementia, dependence, and stroke [1]. The stroke outcome is not yet available. Standard methods have been developed for analysis of each of the other three outcomes.

Cohorts at risk for defined outcomes

1. Mortality cohort

The cohort at risk for the mortality cohort is defined as all those who participated in the baseline survey. More explicitly this includes those who were identified as eligible in the community door-knocking, consented to participate, and completed at least some of the baseline interviews and assessments.

Table 1 – The mortality cohort, at risk at baseline

Site	Number at risk
Cuba	2944 (18.4%)
Dominican Republic	2011 (12.5%)
Puerto Rico	1381 (8.6%)
Peru (urban)	552 (3.4%)
Peru (rural)	1965 (12.3%)
Venezuela	1003 (6.3%)
Mexico (urban)	1000 (6.2%)
Mexico (rural)	1160 (7.2%)
China (urban)	1002 (6.3%)
China (rural)	1005 (6.3%)
India (urban)	2009 (12.5%)
Total	16,032 (100%)

2. Dependence cohort.

Needs for care were rated at baseline, as per Table 2 below

Table 2 – The dependence cohort at risk at baseline

Needs for care	Number (%)
Needs care much of the time	CELL A<br>808 (5.6%)
Needs care some of the time	CELL B<br>662 (4.6%)
Does not need care; they are able to do everything for themselves	CELL C<br>13,049 (89.9%)
Total	14,519 (100%)

The cohort at risk of incident dependence comprises those who were rated as not needing care at baseline (CELL C). Incident dependence was defined as a rating of needing care some or much of the time at the incidence wave follow-up

3. Dementia cohort

Dementia was diagnosed at baseline using the 10/66 dementia algorithm, and the DSM-IV diagnostic criteria. Almost all (94.1%) DSM-IV cases were also identified as cases according to the 10/66 dementia algorithm, but most (60.4%) of 10/66 dementia cases did not meet DSM-IV criteria (Table 3). These were mostly of mild severity. Therefore, incident dementia can be studied according to three outcomes (see Table 3)

a. 10/66 dementia diagnosis (recommended for most purposes). The cohort at risk for the analysis of 10/66 dementia incidence comprises those free of ‘any dementia’ (DSM-IV or 10/66 dementia – Cell A) at baseline
b. DSM-IV dementia. Only those with DSM-IV dementia at baseline are excluded from the cohort at risk when estimating DSM-IV dementia incidence. The cohort at risk comprises Cells A and B
c. ‘any dementia’ (either DSM-IV dementia, or 10/66 dementia, or both). The cohort at risk for the analyses of ‘any dementia’ incidence comprises those free of ‘any dementia’ at baseline (Cell A)

Table 3 – The dementia cohorts at risk at baseline

	10/66 non case	10/66 case	Total
DSM-IV non-case	Cell A<br>13,536 (99.9%)	Cell B<br>779 (60.4%)	14,315 (96.4%)
DSM-IV case	Cell C<br>32 (0.2%)	Cell D<br>510 (39.6%)	542 (3.6%)
Total	13,568 (100%)	1289 (100%)	14,857 (100%)

Follow-up – who from the cohort at risk can be included in the incidence analysis, and how are the person-years calculated?

We made at least five attempts to trace each baseline participant at follow-up, and secure consent for a repeat assessment. Each baseline participant's status at follow-up was coded as

1. reinterviewed (date of interview recorded)

2. deceased (date of death recorded)

3. refused

4. could not be traced, or

5. could not be contacted (reputed to be alive, but could not be contacted for reinterview)

For the mortality cohort vital status at follow-up was ascertained for groups 1-3 (reinterviewed, deceased or refused), as those who refused follow-up interview were alive at that time. Dates of death, and dates of follow-up interview were recorded exactly. Since the date of refusal was not recorded, it was estimated as the median date of follow-up interview in that site. Vital status could not be ascertained with confidence among those who were not traced or could not be contacted, and these participants were excluded from cohort analysis.

For the dependence and dementia cohorts, outcome status at follow-up could be ascertained for those who were reinterviewed (group 1), and for most of those who had died (group 2), through a detailed verbal autopsy interview covering the period leading up to the older person's death. Further details of the methods used to allocate ante-mortem outcome status (probable incident dementia [2] and dependence [3]) are provided elsewhere. Dates of onset of dependence and dementia could not be determined accurately, and hence were assumed to have occurred at the midpoint between baseline interview and reinterview, or death (for those with incidence before death ascertained through verbal autopsy).

Dependence and dementia outcomes could not be ascertained with confidence among those who refused interview, who were not traced or could not be contacted, and these participants are excluded from cohort analysis.

Further details of availability for analysis, and calculation of person-years at risk contributed for each cohort, by status at follow-up are provided in Table 4 below

Table 4 - Availability for analysis, and calculation of person-years at risk contributed for each cohort, by status at follow-up

Cohort	Mortality		Dependence		Dementia	
FU status	Analysis	Person years	Analysis	Person years	Analysis	Person years
Reinterviewed	Included (Outcome - Alive)	Interval between baseline and follow-up interviews	Included	Incident dependence: Half of the interval between baseline and follow-up interviews	Included	Incident dementia: Half of the interval between baseline and follow-up interviews
				No incident dependence: Interval between baseline and follow-up interviews		No incident dementia: Interval between baseline and follow-up interviews
Deceased	Included (Outcome - Deceased)	Interval between baseline interview and date of death	Included if verbal autopsy available to determine incidence of dependence before death, or dependence-free death (competing risk)	Incident dependence: Half of the interval between baseline interview and date of death	Included if verbal autopsy available to determine incidence of dementia before death, or dementia-free death (competing risk)	Incident dementia: Half of the interval between baseline interview and date of death
				Dependence free death: Interval between baseline and date of death		Dementia free death: Interval between baseline and date of death

Refused	Included (Outcome - Alive)	Interval between baseline interview and median date of interviews in that site	Excluded	N/A	Excluded	N/A
Not traced	Excluded	N/A	Excluded	N/A	Excluded	N/A
Not contacted	Excluded	N/A	Excluded	N/A	Excluded	N/A

Analysis plan

For each cohort analysis, we report attrition from the cohort at risk by reason for loss to follow-up, clarifying those who could, and could not be included in the final analysis. We report the relevant characteristics of the cohort at risk at baseline, and any differences between those who could and could not be included in the final analysis (that might contribute to attrition bias).

For descriptive purposes, age-specific incidence (with Poisson standard errors and 95% confidence intervals) is estimated for each site and/ or country, by sex and by age in 5-year bands by dividing numbers of cases by the person-years contributed in each age band. We used direct standardization (applying age-specific incidence rates from each 10/66 site to person-years from the EURODEM multisite European incidence study (21)) to compare rates between 10/66 sites and with those previously observed in EURODEM. We used direct standardization (with the whole 10/66 incidence sample as the standard population) to compare rates of 10/66 dementia between sites, standardizing for age, sex, education and assets.

We model the effects of covariates on mortality using Cox's proportional hazards regression, and the effect of covariates on the incidence of dementia and dependence using competing-risks regression derived from Fine and Gray's proportional subhazards model [4] Stata stcrreg command). Competing-risk regression is based on a cumulative incidence function, indicating the probability of failure (dementia or dependence onset) before a given time, acknowledging the possibility of a competing event (dementia- or dependence-free death). In conventional Cox's proportional hazards regression, deaths are right-censored with such individuals treated as no more or less likely to fail from the cause of interest than those still at risk. This is inappropriate because, after death, failure from dementia or dependence is not merely unobservable, but no longer possible. Competing risks regression works by keeping those who experience competing events at risk so that they can be counted as having no chance of failing.

The base model for all regressions includes the mutually adjusted independent effects of age (per five-year band), sex (men compared with women), and education (per level). Other covariates are added to this model to control for confounding, and test for a priori hypothesized interactions, depending upon the hypothesized exposure of interest. We estimate and report effect sizes with robust 95% confidence intervals to take account of household clustering, for each site and/ or country separately. We then use fixed effects meta-analysis to combine them. Higgins I^2 is computed, estimating the proportion of between-site variability in the estimates accounted for by heterogeneity, as opposed to

sampling error; up to 40% heterogeneity is conventionally considered negligible, while up to 60% may reflect moderate heterogeneity [5]. The assumption underlying a fixed effect meta-analysis is that the underlying true effect is the same across all sites, and any variation is accounted for by sampling error. In the event of extreme heterogeneity, with suggestive trends of effect sizes across sites, exploratory meta-regressions may be performed. These are likely to be underpowered, and should be regarded as hypothesis-generating, at best.

REFERENCES

1. Prina AM, Acosta D, Acostas I, Guerra M, Huang Y, Jotheeswaran AT, et al. Cohort Profile: The 10/66 study. IntJEpidemiol. 2016; dyw056.

2. Prince M, Acosta D, Ferri CP, Guerra M, Huang Y, Rodriguez JJ, et al. Dementia incidence and mortality in middle-income countries, and associations with indicators of cognitive reserve: a 10/66 Dementia Research Group population-based cohort study. Lancet. 2012.

3. Bao J, Chua K-C, Prina M, Prince M. Multimorbidity and care dependence in older adults: a longitudinal analysis of findings from the 10/66 study. BMC Public Health. 2019;19: 585. doi:10.1186/s12889-019-6961-4

4. Fine JP, Gray RJ. A proportional hazards model for the subdistribution of a competing risk. J Am Stat Assoc. 1999;94: 496–509.

5. Higgins JP, Thompson SG. Quantifying heterogeneity in a meta-analysis. StatMed. 2002;21: 1539–1558.

Bibliography of 10/66 Dementia Research Group cohort analyses

1. Prince M, Acosta D, Ferri CP, Guerra M, Huang Y, Rodriguez JJ, et al. Dementia incidence and mortality in middle-income countries, and associations with indicators of cognitive reserve: a 10/66 Dementia Research Group population-based cohort study. Lancet. 2012.

2. Ferri CP, Acosta D, Guerra M, Huang Y, Llibre-Rodriguez JJ, Salas A, et al. Socioeconomic factors and all cause and cause-specific mortality among older people in Latin America, India, and China: a population-based cohort study. PLoSMed. 2012;9: e1001179.

3. Jotheeswaran AT, Williams JD, Prince MJ. Predictors of mortality among elderly people living in a south Indian urban community; a 10/66 Dementia Research Group prospective population-based cohort study. BMCPublic Health. 2010;10: 366.

4. Jotheeswaran AT, Bryce R, Prina M, Acosta D, Ferri CP, Guerra M, et al. Frailty and the prediction of dependence and mortality in low- and middle-income countries: a 10/66 population-based cohort study. BMCMed. 2015;13:138. doi: 10.1186/s12916-015-0378–4.: 138–0378.

5. Guerchet MM, Guerra M, Huang Y, Lloyd-Sherlock P, Sosa AL, Uwakwe R, et al. A cohort study of the effects of older adult care dependence upon household economic functioning, in Peru, Mexico and China. PloS One. 2018;13: e0195567. doi:10.1371/journal.pone.0195567

6. Prince MJ, Acosta D, Guerra M, Huang Y, Jimenez-Velazquez IZ, Llibre Rodriguez JJ, et al. Leg length, skull circumference, and the incidence of dementia in Latin America and China: A 10/66 population-based cohort study. PloS One. 2018;13: e0195133. doi:10.1371/journal.pone.0195133

7. Prince MJ, Acosta D, Guerra M, Huang Y, Jimenez-Velazquez IZ, Llibre Rodriguez JJ, et al. Reproductive period, endogenous estrogen exposure and dementia incidence among women in Latin America and China; A 10/66 population-based cohort study. PloS One. 2018;13: e0192889. doi:10.1371/journal.pone.0192889

8. Bao J, Chua K-C, Prina M, Prince M. Multimorbidity and care dependence in older adults: a longitudinal analysis of findings from the 10/66 study. BMC Public Health. 2019;19: 585. doi:10.1186/s12889-019-6961-4

9. Prina AM, Stubbs B, Veronese N, Guerra M, Kralj C, Llibre Rodriguez JJ, et al. Depression and Incidence of Frailty in Older People From Six Latin American Countries. Am J Geriatr Psychiatry Off J Am Assoc Geriatr Psychiatry. 2019;27: 1072–1079. doi:10.1016/j.jagp.2019.04.008

10. Cherbuin N, Walsh EI, Prina AM. Chronic Obstructive Pulmonary Disease and Risk of Dementia and Mortality in Lower to Middle Income Countries. J Alzheimers Dis JAD. 2019;70: S63–S73. doi:10.3233/JAD-180562

11. Daskalopoulou C, Prince M, Koukounari A, Haro JM, Panagiotakos DB, Prina AM. Healthy ageing and the prediction of mortality and incidence dependence in low- and middle- income countries: a 10/66 population-based cohort study. BMC Med Res Methodol. 2019;19: 225. doi:10.1186/s12874-019-0850-5

12. Johansson L, Guerra M, Prince M, Horder H, Falk H, Stubbs B, et al. Associations between Depression, Depressive Symptoms, and Incidence of Dementia in Latin America: A 10/66 Dementia Research Group Study. J Alzheimers Dis JAD. 2019;69: 433–441. doi:10.3233/JAD-190148

13. Pasquini L, Llibre Guerra J, Prince M, Chua K-C, Prina AM. Neurological signs as early determinants of dementia and predictors of mortality among older adults in Latin America: a 10/66 study using the NEUROEX assessment. BMC Neurol. 2018;18: 163. doi:10.1186/s12883-018-1167-4

14. Wu Y-T, Kralj C, Acosta D, Guerra M, Huang Y, Jotheeswaran AT, et al. The association between, depression, anxiety, and mortality in older people across eight low- and middle-income countries: Results from the 10/66 cohort study. Int J Geriatr Psychiatry. 2020;35: 29–36. doi:10.1002/gps.5211
