# Supplementary material for: Intrinsic capacity and its associations with incident dependence and mortality in 10/66 Dementia Research Group studies in Latin America, India, and China: A population-based cohort study
Source: PLoS Med. 2021 Sep 14;18(9):e1003097. doi: 10.1371/journal.pmed.1003097 (PMC8439485; doi:10.1371/journal.pmed.1003097)
Supplement: S2 Appendix — (PDF) [file pmed.1003097.s002.pdf]

S2 APPENDIX
ADDITIONAL ANALYSES SUGGESTED BY REVIEWERS

We are grateful to the reviewers for suggesting two additional sets of analyses to clarify further findings presented in the main paper. The findings from these analyses have been summarised in the main paper, and detailed methods and results are presented in this supplementary file.

SUMMARY OF CONTENTS

SECTION 1
A SUMMARY OF PREVALENCE OF RETAINED CAPACITY FOLLOWING DIRECT STANDARDIZATION FOR AGE AND GENDER

Page	Table	Title
2	1.1	Crude and standardised prevalence of neuromuscular capacity by site
2	1.2	Crude and standardised prevalence of vitality (nutrition)capacity by site
3	1.3	Crude and standardised prevalence of vision capacity by site
3	1.4	Crude and standardised prevalence of hearing capacity by site
3	1.5	Crude and standardised prevalence of cognitive capacity by site
3	1.6	Crude and standardised prevalence of psychological capacity by site
4	1.7	Crude and standardised prevalence of continence capacity by site
4	1.8	Crude and standardised prevalence of full capacity retained (all domains) by site

SECTION 2
ATTRITION IN THE MORTALITY AND DEPENDENCE COHORTS

Page	Table	Title
5	2.1	Mortality Cohort – outcome status at follow-up, by site
7	2.2	Adjusted associations of sociodemographic and health characteristics with attrition in the mortality cohort (vital status not ascertained at follow-up)
8	2.3	Predicted probability of death[1] before follow-up compared between groups, according to vital status ascertainment
10	2.4	Dependence Cohort - outcome status at follow-up, by site
11	Fig 2.1	Cohort Flow Chart – Dependence Cohort
13	2.5	Baseline sociodemographic and health characteristics of the dependence cohort, by status at follow-up
14	2.6	Adjusted associations of sociodemographic and health characteristics with loss to follow-up in the dependence cohort

SECTION 1

A SUMMARY OF PREVALENCE OF RETAINED CAPACITY FOLLOWING DIRECT STANDARDIZATION FOR AGE AND GENDER

We have reported quite extensive variation among sites in the prevalences of retained intrinsic capacity (for each domain, and for full capacity retained in all domains) (main paper, Table 2). However, age was an important determinant of prevalence of retained intrinsic capacity (main paper, Figs 1 and 2) and the demographic composition of the catchment area samples was also quite variable (main paper, Table 1). In general, mean ages and the proportion of women were lower in rural and less developed sites. Mean age varied from 71.3 years (urban India) to 76.3 years (Puerto Rico), and the proportion of women from 54.6% (rural India) to 76.3% (Puerto Rico). A reviewer therefore suggested that we should standardize the prevalences of retained capacity for age and gender to permit a comparison accounting for the different demographic compositions of the baseline wave samples among the catchment area sites. Accordingly, we carried out direct standardization by age group (in five-year bands) and gender, using the pooled sample across all 12 sites as the standard population, and the Stata dstdize command.

The results of these analyses are displayed in Tables 1.1 to 1.8 below, as prevalence proportions rather than percentages, with the crude prevalence (Crude) and the prevalence standardised for age and gender (Adj. Rate) and its 95% confidence intervals. The standardized prevalences are very similar to the crude prevalences throughout, from which it can be concluded that compositional differences in age and gender between the catchment area samples cannot account for the sizeable differences in the prevalence of retained capacity observed among sites.

TABLE 1.1 CRUDE AND STANDARDISED PREVALENCE OF NEUROMUSCULAR CAPACITY BY SITE

Centre	N	Crude	Adj_Rate	Confidence Interval	
Cuba	2914	0.586479	0.607270	[0.590516,	0.624025]
Dominican Rep	1917	0.420449	0.437491	[0.415650,	0.459332]
Puerto Rico	1516	0.849604	0.871678	[0.855865,	0.887492]
Peru (urban)	1372	0.651603	0.667832	[0.643993,	0.691672]
Peru (rural)	550	0.747273	0.744189	[0.707272,	0.781105]
Venezuela	1456	0.815934	0.788018	[0.766459,	0.809578]
Mexico (urban)	940	0.939362	0.937925	[0.921979,	0.953871]
Mexico (rural)	913	0.935378	0.933662	[0.917551,	0.949773]
China (urban)	1160	0.880172	0.874828	[0.855957,	0.893698]
China (rural)	998	0.338677	0.310059	[0.281642,	0.338476]
India (urban)	979	0.910112	0.896685	[0.875193,	0.918177]
India (rural)	999	0.907908	0.890394	[0.868987,	0.911801]

TABLE 1.2 CRUDE AND STANDARDISED PREVALENCE OF VITALITY (NUTRITION) CAPACITY BY SITE

Centre	N	Crude	Adj_Rate	Confidence Interval	
Cuba	2930	0.878840	0.884644	[0.873358,	0.895931]
Dominican Rep	1991	0.836765	0.841667	[0.825707,	0.857627]
Puerto Rico	1611	0.899441	0.904953	[0.890302,	0.919603]
Peru (urban)	1370	0.814599	0.816565	[0.796091,	0.837039]
Peru (rural)	551	0.805808	0.800598	[0.766656,	0.834541]
Venezuela	1583	0.817435	0.808878	[0.788752,	0.829005]
Mexico (urban)	994	0.890342	0.890459	[0.870662,	0.910256]
Mexico (rural)	991	0.810293	0.809636	[0.785368,	0.833903]
China (urban)	1154	0.984402	0.984106	[0.976701,	0.991511]
China (rural)	998	0.986974	0.986218	[0.978369,	0.994066]
India (urban)	977	0.663255	0.644653	[0.612055,	0.677252]
India (rural)	979	0.660878	0.648579	[0.616790,	0.680368]

TABLE 1.3 CRUDE AND STANDARDISED PREVALENCE OF VISION CAPACITY BY SITE

Centre	N	Crude	Adj_Rate	Confidence Interval	
Cuba	2924	0.703830	0.712600	[0.696450,	0.728750]
Dominican Rep	2003	0.596605	0.605415	[0.583983,	0.626847]
Puerto Rico	1935	0.741602	0.758258	[0.738854,	0.777663]
Peru (urban)	1374	0.668850	0.676423	[0.652032,	0.700814]
Peru (rural)	551	0.638838	0.640005	[0.599120,	0.680890]
Venezuela	1908	0.599581	0.587872	[0.564936,	0.610809]
Mexico (urban)	998	0.713427	0.714257	[0.685806,	0.742708]
Mexico (rural)	991	0.642785	0.643353	[0.613551,	0.673155]
China (urban)	1160	0.829310	0.826791	[0.804694,	0.848887]
China (rural)	998	0.935872	0.928599	[0.911040,	0.946157]
India (urban)	986	0.910751	0.901720	[0.881171,	0.922268]
India (rural)	999	0.776777	0.760278	[0.732038,	0.788518]

TABLE 1.4 CRUDE AND STANDARDISED PREVALENCE OF HEARING CAPACITY BY SITE

Centre	N	Crude	Adj_Rate	Confidence Interval	
Cuba	2927	0.894773	0.901243	[0.890926,	0.911560]
Dominican Rep	2003	0.826760	0.832594	[0.816428,	0.848760]
Puerto Rico	1934	0.832472	0.845005	[0.828779,	0.861230]
Peru (urban)	1374	0.778020	0.787033	[0.765985,	0.808080]
Peru (rural)	551	0.829401	0.836766	[0.805840,	0.867692]
Venezuela	1915	0.845431	0.833089	[0.815329,	0.850850]
Mexico (urban)	998	0.798597	0.797739	[0.772655,	0.822823]
Mexico (rural)	992	0.769153	0.771768	[0.746086,	0.797451]
China (urban)	1160	0.873276	0.873457	[0.854576,	0.892338]
China (rural)	998	0.908818	0.893916	[0.872869,	0.914962]
India (urban)	985	0.967513	0.963802	[0.950508,	0.977095]
India (rural)	999	0.844845	0.816211	[0.790519,	0.841904]

TABLE 1.5 CRUDE AND STANDARDISED PREVALENCE OF COGNITIVE CAPACITY BY SITE

Centre	N	Crude	Adj_Rate	Confidence Interval	
Cuba	2935	0.777853	0.792963	[0.779387,	0.806539]
Dominican Rep	2005	0.699252	0.721324	[0.702774,	0.739874]
Puerto Rico	1935	0.769509	0.797397	[0.780029,	0.814765]
Peru (urban)	1378	0.846880	0.858694	[0.842296,	0.875092]
Peru (rural)	552	0.789855	0.779647	[0.745092,	0.814201]
Venezuela	1928	0.834544	0.807063	[0.789013,	0.825112]
Mexico (urban)	998	0.729459	0.737814	[0.712427,	0.763201]
Mexico (rural)	992	0.620968	0.622999	[0.593724,	0.652273]
China (urban)	1160	0.903448	0.895396	[0.877806,	0.912986]
China (rural)	998	0.821643	0.783521	[0.756305,	0.810738]
India (urban)	986	0.570994	0.531182	[0.499465,	0.562900]
India (rural)	999	0.332332	0.285417	[0.261272,	0.309561]

TABLE 1.6 CRUDE AND STANDARDISED PREVALENCE OF PSYCHOLOGICAL CAPACITY BY SITE

Centre	N	Crude	Adj_Rate	Confidence Interval	
Cuba	2885	0.763951	0.768117	[0.752935,	0.783300]
Dominican Rep	1992	0.619980	0.629029	[0.607987,	0.650072]
Puerto Rico	1902	0.827024	0.826491	[0.808699,	0.844282]
Peru (urban)	1335	0.707865	0.712254	[0.688219,	0.736289]
Peru (rural)	548	0.737226	0.725339	[0.686916,	0.763762]
Venezuela	1920	0.708854	0.703537	[0.682352,	0.724723]
Mexico (urban)	993	0.688822	0.697713	[0.669189,	0.726237]
Mexico (rural)	984	0.740854	0.737754	[0.710608,	0.764900]
China (urban)	1126	0.960924	0.959596	[0.947737,	0.971456]
China (rural)	974	0.984600	0.984990	[0.977205,	0.992775]
India (urban)	982	0.609980	0.596131	[0.563237,	0.629025]
India (rural)	954	0.557652	0.556212	[0.522576,	0.589847]

TABLE 1.7 CRUDE AND STANDARDISED PREVALENCE OF CONTINENCE CAPACITY BY SITE

Centre	N	Crude	Adj_Rate	Confidence Interval	
Cuba	2907	0.961128	0.964847	[0.958561,	0.971133]
Dominican Rep	2003	0.953070	0.957786	[0.949383,	0.966190]
Puerto Rico	1927	0.977686	0.981611	[0.975956,	0.987266]
Peru (urban)	1376	0.957122	0.959944	[0.949962,	0.969927]
Peru (rural)	550	0.987273	0.985825	[0.975270,	0.996380]
Venezuela	1925	0.977143	0.971555	[0.963277,	0.979833]
Mexico (urban)	998	0.973948	0.974197	[0.964480,	0.983915]
Mexico (rural)	992	0.985887	0.986267	[0.979075,	0.993459]
China (urban)	1158	0.949050	0.946466	[0.933183,	0.959749]
China (rural)	998	0.982966	0.980487	[0.970834,	0.990140]
India (urban)	976	0.980533	0.977150	[0.966211,	0.988090]
India (rural)	997	0.980943	0.975115	[0.963411,	0.986820]

TABLE 1.8 CRUDE AND STANDARDISED PREVALENCE OF FULL CAPACITY RETAINED(ALL DOMAINS) BY SITE

Centre	N	Crude	Adj_Rate	Confidence Interval	
Cuba	2935	0.286542	0.301199	[0.284982,	0.317416]
Dominican Rep	2005	0.149626	0.159259	[0.143001,	0.175517]
Puerto Rico	1935	0.406718	0.437740	[0.414844,	0.460635]
Peru (urban)	1378	0.278665	0.291659	[0.267987,	0.315332]
Peru (rural)	552	0.259058	0.255800	[0.219418,	0.292182]
Venezuela	1928	0.313797	0.292375	[0.272293,	0.312456]
Mexico (urban)	998	0.342685	0.351399	[0.321878,	0.380921]
Mexico (rural)	992	0.254032	0.255462	[0.228802,	0.282121]
China (urban)	1160	0.627586	0.621657	[0.594698,	0.648615]
China (rural)	998	0.262525	0.228449	[0.203986,	0.252911]
India (urban)	986	0.273834	0.244373	[0.218166,	0.270580]
India (rural)	999	0.120120	0.101427	[0.084653,	0.118202]

SECTION 2
ATTRITION IN THE MORTALITY AND DEPENDENCE COHORTS

In response to reviewer requests, we provide further information on the extent and reasons for attrition in the mortality and dependence cohorts. We also explore potential for attrition bias by estimating the associations of relevant baseline covariates with failure to ascertain vital status at follow-up in the mortality cohort, and with attrition through loss to follow up in the dependence cohort.

At cohort inception, the mortality cohort comprised of 15,901 older people who had participated in the baseline surveys in the six Latin American countries, China and the urban India site. A mortality follow-up was not conducted in the rural India site.

At cohort inception, the dependence cohort comprised of 12,939 older people who had participated in the baseline surveys in in the six Latin American countries and China, and who were assessed not to have needs for care (dependence-free) at baseline. The full follow-up interview was not conducted in either of the India sites.

Attrition in the mortality cohort
Vital status (alive or dead) could be determined for all those who were re-interviewed, were found alive but refused interview, or were confirmed to be dead by family members or other key informants. Vital status could not be confirmed for those who were not traced. Those who were 'uncontactable' were said to be still alive. However, since this could not be definitively confirmed (by contacting the older person concerned), we also excluded them from the analysis.

Table 1 below provides information on attrition in the mortality cohort, by site. Overall vital status was ascertained for 13,949 of the 15,901 participants (87.7%) in the mortality cohort, and not ascertained for 1,952 (12.3%). Vital status could not be confirmed for a relatively higher proportion of older people in certain of the urban sites; in Puerto Rico (21.6%), India (21.6%), Dominican Republic (15.2%), China (14.7%), and Venezuela (13.6%). Older populations in the rural sites were generally more stable and easier to trace.

Table 2.1 – Mortality Cohort – outcome status at follow-up, by site

CENTRE	Total cohort	Found alive	Death confirmed	Vital status ascertained	Vital status not ascertained
Cuba	2813	2027 (72.1%)	608 (21.6%)	2635 (93.7%)	178 (6.3%)
Dominican Republic	2011	1239 (61.6%)	467 (23.2%)	1706 (84.8%)	305 (15.2%)
Puerto Rico	2009	1278 (63.6%)	298 (14.9%)	1576 (78.4%)	433 (21.6%)
Peru urban	1381	1147 (83.1%)	98 (7.1%)	1245 (90.2%)	136 (9.8%)
Peru rural	552	453 (82.1%)	54 (9.8%)	507 (91.8%)	45 (8.2%)
Venezuela	1965	1497 (76.2%)	200 (10.2%)	1697 (86.4%)	268 (13.6%)
Mexico urban	1003	812 (81.0%)	99 (9.9%)	911 (90.8%)	92 (9.2%)
Mexico rural	1000	823 (82.3%)	110 (11.0%)	933 (93.3%)	67 (6.7%)
China urban	1160	765 (65.9%)	224 (19.3%)	989 (85.3%)	171 (14.7%)
China rural	1002	711 (71.0%)	291 (29.0%)	1002 (100%)	0 (0.0%)
India urban	1005	595 (59.2%)	153 (14.9%)	748 (78.4%)	257 (21.6%)
TOTAL	15901	11347 (71.4%)	2602 (16.4%)	13949 (87.7%)	1952 (12.3%)

Local factors may have accounted for relatively high proportions of older people not being traced, or uncontactable in the urban sites; outmigration to the USA from Dominican Republic, and urban regeneration programs that led to the demolition of many homes and displacement of participants in the catchment areas in urban China and India. In the Puerto Rico site reasons for losses to follow-up were inadvertently not recorded for 303 participants (a protocol violation) accounting for much of the failure to ascertain vital status in that site.

In a previous publication (1), we had explored potential for attrition bias in the estimation of mortality by predicting the probability of death in a logistic regression including the following baseline covariates; age, gender, education, WHODAS 2.0 disability score and dementia diagnosis. We then compared predicted probabilities of death for those who did and did not have vital status ascertained at follow-up. That analysis indicated that those who did not have vital status ascertained had higher predicted probabilities of death in urban Mexico and lower predicted probabilities in urban China. However, this analysis did not include data from the Puerto Rico site, which was not complete, and the selected health covariates were not directly relevant to the exposures of interest in the current study.

Therefore to further explore potential for bias in the estimation of associations with incident mortality we modelled the effects of number of DICs and other known or suspected determinants of mortality in these populations (1,2) (age, gender, education, frailty and dependence), at baseline, on attrition. We did this using multivariable logistic regression, with vital status not ascertained at follow-up as the outcome, running models separately for each site, and then pooling the results meta-analytically. Initially we ran a model including the effects of age (per 5-year band), gender, education (per level) and number of DICs. We then substituted frailty, and then dependence for number of DICs to estimate their effects, also controlling for age, gender and education. Rural China was excluded from the analysis, since in that site vital status was ascertained on all participants.

The results are summarized in Table 2.2, below. Age and gender did not appear to predict failure to ascertain vital status at outcome, in any site, or after the effects were pooled. In urban China those with higher levels of education were less likely to have vital status ascertained, but the pooled effect across sites suggested no overall association. The pooled effects of number of DIC, frailty and dependence also suggested no association. However, heterogeneity of effects was moderate to high for these meta-analyses, and there was a suggestion that a greater number of DIC, and frailty might be positively associated with attrition in Puerto Rico, and negatively associated in the Dominican Republic.

Table 2.2 – Adjusted associations[1] of sociodemographic and health characteristics with attrition in the mortality cohort (vital status not ascertained at follow-up)

Baseline covariates<br><br>SITE	Age (in 5 year bands)[2]	Gender (male versus female) [2]	Education (by level) [2]	Number of declines in intrinsic capacity[3]	Frailty[3]	Dependence (any needs for care)[3]
Cuba	1.06 (0.90-1.24)	0.76 (0.55-1.04)	1.18 (1.00-1.39)	0.95 (0.83-1.08)	0.89 (0.58-1.37)	1.03 (0.59-1.80)
Dominican Republic	0.99 (0.88-1.11)	1.04 (0.81-1.33)	1.03 (0.90-1.18)	**0.89 (0.80-0.98)**	**0.68 (0.48-0.94)**	**0.59 (0.37-0.94)**
Puerto Rico	0.97 (0.87-1.08)	1.03 (0.84-1.27)	0.91 (0.82-1.01)	**1.11 (1.00-1.22)**	**1.34 (1.00-1.78)**	1.18 (0.83-1.68)
Peru urban	1.17 (0.97-1.42)	1.12 (0.72-1.74)	0.93 (0.74-1.15)	0.86 (0.75-0.99)	0.73 (0.45-1.20)	0.99 (0.54-1.80)
Peru rural	1.23 (0.95-1.58)	0.91 (0.50-1.65)	0.90 (0.63-1.27)	0.95 (0.76-1.19)	1.18 (0.55-2.55)	DNC
Venezuela	1.07 (0.94-1.22)	0.93 (0.72-1.20)	0.93 (0.79-1.10)	**1.13 (1.01-1.26)**	1.19 (0.81-1.74)	0.69 (0.41-1.15)
Mexico urban	1.06 (0.87-1.30)	1.14 (0.75-1.73)	1.07 (0.85-1.35)	1.20 (0.98-1.46)	0.70 (0.37-1.32)	**2.62 (1.38-4.97)**
Mexico rural	1.06 (0.84-1.33)	1.45 (0.89-2.36)	1.19 (0.87-1.63)	0.91 (0.74-1.12)	0.49 (0.21-1.14)	1.17 (0.49-2.80)
China urban	0.88 (0.74-1.05)	0.79 (0.60-1.03)	**1.21 (1.04-1.42)**	0.87 (0.69-1.08)	1.05 (0.55-1.99)	0.72 (0.41-1.28)
India urban	0.97 (0.84-1.12)	0.94 (0.69-1.28)	1.01 (0.87-1.17)	1.04 (0.90-1.20)	1.30 (0.89-1.92)	1.92 (0.88-4.23)
Pooled fixed effect	1.02 (0.97-1.07)	0.96 (0.88-1.06)	1.01 (0.96-1.07)	0.99 (0.95-1.04)	0.99 (0.87-1.14)	1.00 (0.84-1.20)
Higgins I^2(%)	1 (0-63)	1 (0-63)	44 (0-73)	65 (32-82)	50 (0-76)	61 (19-81)

1. adjusted odds ratios and their 95% confidence intervals, derived from multivariable logistic regression
2. adjusted for other sociodemographic characteristics (age, gender, and education) and number of declines in intrinsic capacity
3. adjusted for age, gender, and education

We then replicated the analysis previously conducted, predicting probability of death, but with a different set of covariates; age, gender, education, number of DIC, frailty and dependence. Findings are summarized in Table 2.3 below. These indicated that those who did not have vital status ascertained had lower predicted probabilities of death in Dominican Republic (where worse health status was inversely associated with attrition) and urban China (where more education was positively associated with attrition.

Table 2.3 Predicted probability of death[1] before follow-up compared between groups, according to vital status ascertainment

Site[2]	Vital status ascertained. Mean predicted probability of death (SD)	Vital status not ascertained. Mean predicted probability of death (SD)	Mean difference	t-value, p-value
Cuba	0.230 (0.190)	0.220 (0.170)	-0.011 (-0.040 to +0.019)	-0.71, 0.48
Dominican Republic	0.271 (0.165)	0.246 (0.143)	-0.024 (-0.044 to -0.004)	-2.40, 0.02
Puerto Rico	0.172 (0.145)	0.187 (0.150)	+0.015 (-0.001 to +0.031	1.90,0.06
Peru (urban)	0.078 (0.116)	0.077 (0.101)	-0.002 (-0.031 to + 0.027)	-0.15, 0.89
Peru (rural)	0.106 (0.097)	0.104 (0.067)	+0.007 (-0.025 to +0.039)	0.13, 0.66
Venezuela	0.109 (0.096)	0.119 (0.114)	+0.010 (-0.002 to +0.023)	1.60, 0.11
Mexico (urban)	0.108 (0.087)	0.128 (0.103)	+0.020 (-0.004 to +0.045)	1.63, 0.11
Mexico (rural)	0.115 (0.087)	0.115 (0.103)	+0.000 (-0.021 to +0.022)	0.04, 0.97
China (urban)	0.226 (0.201)	0.181 (0.167)	-0.046 (-0.074 to -0.018)	-3.20, 0.002
India	0.196 (0.123)	0.194 (0.114)	-0.002 (-0.019 to +0.016)	-0.20, 0.84

1. From a model including the following baseline covariates - age (per 5-year band), gender, education per level), number of declines in intrinsic capacity, frailty, and dependence

2. Vital status was ascertained for all participants in the rural China site

Conclusion – attrition in the mortality cohort

Overall, these analyses provide some reassurance that attrition in the mortality cohort, is relatively modest, may be random with respect to baseline covariates that are known or hypothesized to be associated with mortality, and that the mortality experience of those who did and did not have vital status ascertained was likely to be similar. However, this may not be true of all sites. Local factors might have interacted with health and sociodemographic status to account for some of the heterogeneity in the associations observed. For example, if migration to the USA was an important mechanism in Dominican Republic, impaired and frail older people may have been less likely to move away. The composition of the group for whom vital status was not ascertained differs in Puerto Rico from those in other sites (in that they would almost certainly have included refusals, who may have had worse health, in addition to those who were uncontactable and not traced).

Displacement by urban regeneration in Beijing may have disproportionately affected lower educated households.

Attrition bias in the estimation of associations of DIC, frailty and dependence with mortality cannot be absolutely excluded, and the likely direction is difficult to predict. However, the scope for bias is limited, and it is unlikely to have been of sufficient degree to have altered substantially the main findings from the analysis.

Attrition in the dependence cohort

Attrition in the dependence cohort is both more complex, and more extensive than in the mortality cohort. Dependence status (needs for care) could be determined, in principle, for all those who were re-interviewed from the rating completed during the interview, and for those who had died from data collected in the post mortem verbal autopsy interview with a key informant. However, there was some missing data from both sources, and those with missing outcome data could not contribute to the analysis. Those who refused, were uncontactable or not traced (that is alive or vital status not known, and not re-interviewed) were lost to follow-up (LTFU), and these individuals could also not be included in the analysis due to missing outcome data.

Attrition in the dependence cohort is summarised in Table 2.4, by site and overall.

Those who could be included in the analysis are further broken down into
1. those re-interviewed and rated for incident dependence by the interviewer
2. those deceased and with incident dependence predicted from data collected in the verbal autopsy interview

Those who could not be included in the analysis are further broken down into
1. those re-interviewed but with missing data on the dependence outcome
2. those deceased but with missing or insufficient data from the verbal autopsy to predict the dependence outcome
3. those LTFU (refused, uncontactable, not traced, or reason not provided)

Attrition is also summarised schematically in a cohort flow chart (Figure 2.1).

Overall 10,326 older people from the original cohort of 12,939 (79.8%) could be included in the analysis. Attrition was mainly accounted for by losses to follow-up (n=2,202, 17.0%), with a much smaller contribution from missing outcome data from those re-interviewed (n=55, 0.4%), and missing outcome data from verbal autopsy interviews of the deceased (n=356, 2.8%). In most sites around 80% could be included in the analysis, with around 20% attrition. However, attrition was higher in urban Peru (30.7% - high refusal rate), Venezuela (26.1% - high refusal and uncontactable rates), and negligible in rural China (0.1%) where all participants were followed up and dependence outcome data was missing on just one re-interviewed participant.

Table 2.4 - Dependence Cohort - outcome status at follow-up, by site

CENTRE	Total cohort	Included in the analysis			Not included in the analysis							
		Assessed, and outcome recorded			Assessed but outcome not recorded		Lost to follow-up					Total not included in the analysis
		Re-interviewed	Dead, outcome from verbal autopsy	Total included in the analysis	Re-interviewed but outcome not recorded	Dead, no outcome from verbal autopsy	Refused	Uncontactable	Not traced	Not recorded	Total lost to follow-up	
Cuba	2219	1654 (74.5%)	116 (5.2%)	1770 (79.8%)	19 (0.9%)	270 (12.2%)	12 (0.5%)	108 (4.9%)	40 (1.8%)	0	160 (7.2%)	449 (20.2%)
Dominican Republic	1768	1091 (61.7%)	345 (19.5%)	1436 (81.2%)	10 (0.6%)	0	39 (2.2%)	51 (2.9%)	232 (13.1%)	0	322 (18.2%)	332 (18.8%)
Puerto Rico	1708	1150 (67.3%)	175 (10.2%)	1325 (77.6%)	20 (1.2%)	1 (0.1%)	5 (0.3%)	11 (0.6%)	45 (2.6%)	301 (17.6%)	362 (21.2%)	383 (22.4%)
Peru urban	1246	830 (66.6%)	34 (2.7%)	864 (69.3%)	1 (0.1%)	18 (1.4%)	241 (19.3%)	51 (4.1%)	71 (5.7%)	0	363 (66.7%)	382 (30.7%)
Peru rural	524	399 (76.1%)	44 (8.4%)	443 (84.5%)	4 (0.8%)	1 (0.2%)	32 (6.1%)	25 (4.8%)	19 (3.6%)	0	76 (14.5%)	81 (15.5%)
Venezuela	1744	1153 (66.1%)	136 (7.8%)	1289 (73.9%)	0	2 (0.1%)	215 (12.3%)	232 (13.3%)	0	6 (0.3%)	453 (26.0%)	455 (26.1%)
Mexico urban	889	688 (77.4%)	41 (4.6%)	729 (82.0%)	0	32 (3.6%)	57 (6.4%)	56 (6.3%)	15 (1.7%)	0	128 (14.4%)	160 (18.0%)
Mexico rural	916	664 (72.5%)	53 (5.8%)	717 (78.3%)	0	32 (3.5%)	106 (11.6%)	42 (4.6%)	19 (2.1%)	0	167 (18.2%)	199 (21.7%)
China urban	977	671 (68.7%)	135 (13.8%)	806 (82.5%)	0	0	19 (1.9%)	2 (0.2%)	150 (15.4%)	0	171 (17.5%)	171 (17.5%)
China rural	948	698 (73.6%)	249 (26.3%)	947 (99.9%)	1 (0.1%)	0	0	0	0	0	0	1 (0.1%)
TOTAL	12939	8998 (69.5%)	1328 (10.3%)	10326 (79.8%)	55 (0.4%)	356 (2.8%)	726 (5.6%)	578 (4.5%)	591 (4.6%)	307 (2.4%)	2202 (17.0%)	2613 (20.2%)

Figure 2.1 – Cohort Flow Chart – Dependence Cohort

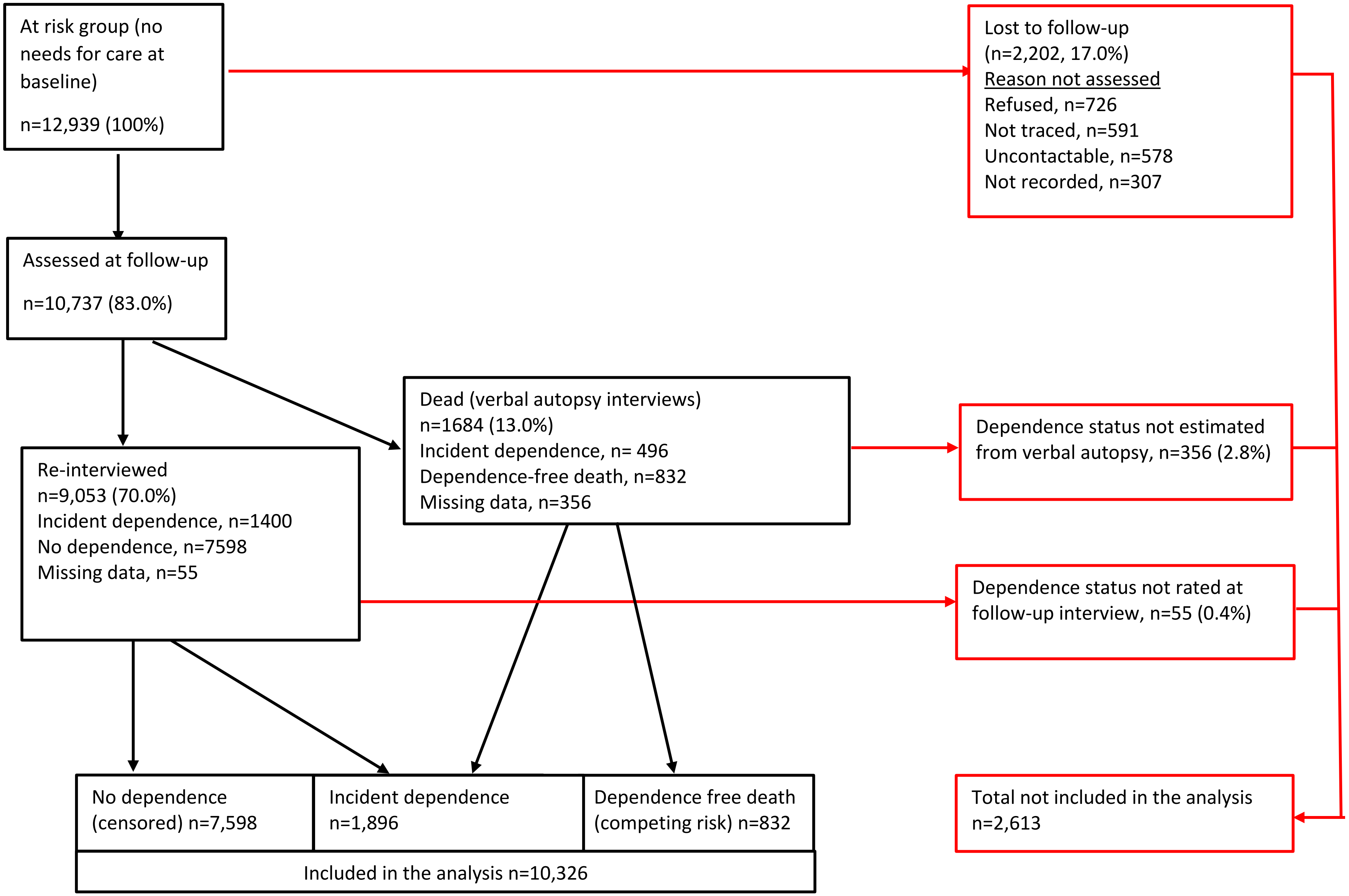

As a first step in understanding patterns of association between baseline covariates and status at follow-up, we compared the characteristics of the three main groups in the dependence cohort by follow-up status, those who were re-interviewed (n=9,053), those who were LTFU (n=2,202), and those who were known to have died n=1,684). The characteristics studied were baseline sociodemographic and health covariates that might be determinants of risk for incident dependence. These comprise age, gender and education, cognitive function (the Community Screening Interview for Dementia – CSID (3)), multimorbidity (defined as two or more chronic disease diagnoses (4)), frailty (2), disability (15 or more disability days in the last month), and DIC. The descriptive and univariate analyses were conducted on data pooled across sites. For the dichotomous variables (male gender, low education, multimorbidity, frailty, disability and one or more DIC we compared proportions exposed, using Chi squared tests for heterogeneity with a null hypothesis of no difference in exposure proportions across the three groups. For continuously distributed variables (age, COGSCORE, number of DIC) we compared means, using one-way ANOVA with a null hypothesis of no difference in means across the three groups. Contrasts (mean differences with 95% confidence intervals) are provided for LTFU vs re-interviewed, and dead vs re-interviewed. Given the non-normal distribution of number of DIC we also used Kruskall Wallis non-parametric one-way ANOVA comparing ranks across the three groups.

Findings are presented in Table 2.5 below. There is substantial and statistically significant heterogeneity among the three groups for all characteristics studied, other than multimorbidity. Those who had died were older, had poorer cognitive performance, and were more likely to be male, less educated, frail, to have experienced significant disability, and to have more DICs at baseline. However, those who were LTFU were more akin to those who were re-interviewed in all these respects. They were still significantly older, more cognitively impaired and affected by a greater number of DIC than those who were re-interviewed, but the differences were negligible, and much smaller than those between the deceased and re-interviewed groups.

Table 2.5 - Baseline demographic and health characteristics of the dependence cohort, by status at follow-up

	Re-interviewed (n=9,053)	Lost to follow-up (n=2,202)	Dead (n=1,684)	p-value
Age, mean (SD)	73.1 (6.2)	73.8 (6.7)<br>+0.7 (+0.3 to +1.1)[1]	77.3 (7.4)<br>+4.2 (+3.8 to +4.6) [1]	<0.001[2]
Male gender	3249 (35.9%)	808 (36.7%)	799 (47.4%)	<0.001[3]
Education (did not complete primary)	3491 (38.7%)	776 (35.3%)	869 (51.8%)	<0.001[3]
Cognitive function (COGSCORE), mean (SD)	30.3 (2.8)	30.1 (2.7)<br>-0.2 (-0.4 to 0.0) [1]	29.1 (3.9)<br>-1.2 (-1.4 to -1.0) [1]	<0.001[2]
Multimorbidity	2284 (25.2%)	578 (26.3%)	462 (27.4%)	0.13[3]
Frailty	903 (10.0%)	252 (11.4%)	320 (19.4%)	<0.001
>15 disability days in last month	834 (9.7%)	211 (10.2%)	232 (14.1%)	<0.001[3]
One or more DIC	5807 (64.1%)	1434 (65.1%)	1283 (76.2%)	<0.001[3]
Number of domains affected by DIC				
Median (1st and 3rd quartiles)	1 (0,2)	1 (0,2)	1 (0,2)	<0.001[4]
Mean (SD)	1.14 (1.16)	1.18 (1.17)<br>+0.04 (-0.02 to +0.11) [1]	1.61 (1.36)<br>+0.47 (+0.39 to +0.54) [1]	<0.001[2]

1. Mean difference (contrast with re-interviewed group), and 95% confidence intervals
2. ANOVA, test for heterogeneity among groups
3. Chi squared test for heterogeneity among groups
4. Kruskall- Wallis test for heterogeneity among groups

To further explore potential for bias in the estimation of associations with incident dependence we followed a similar approach to that used in the mortality cohort, modelling the effects of number of DICs and other known or suspected determinants of incident dependence (age, gender, education, and frailty) at baseline on attrition. We did this using multivariable logistic regression, with LTFU as the outcome and those who were re-interviewed as the reference category. Those who were known to have died were omitted from this analysis. We ran models separately for each site, and then pooled the results meta-analytically. Initially we ran a model including the effects of age (per 5-year band), gender, education (per level) and number of DICs. We then substituted frailty for number of DICs to estimate its effect, also controlling for age, gender and education. Rural China was excluded from the analysis, since in that site only one participant was LTFU.
Results are summarized in Table 2.6 below. The only consistent independent association across sites was that between older age and LTFU (aOR 1.07, 95% CI 1.02-1.12, per 5-year increment in age). Number of DIC was positively associated with LTFU in Puerto Rico, and inversely associated in rural Mexico, but with no overall effect across sites (pooled aOR 0.98, 95% CI 0.94-1.03). There was also no evidence that frailty predicted LTFU, when we substituted this exposure for number of DIC in the model (pooled aOR 1.06, 95% CI 0.91-1.23).

Table 2.6
Adjusted associations[1] of sociodemographic characteristics and baseline declines in intrinsic capacity with loss to follow-up

Baseline covariate<br><br>SITE	Age (in 5 year bands) [2]	Gender (male versus female) [2]	Education (by level) [2]	Number of declines in intrinsic capacity[3]	Frailty[3]
Cuba	1.17 (0.99-1.39)	0.85 (0.60-1.18)	1.19 (1.00-1.43)	1.08 (0.92-1.26)	1.18 (0.71-1.95)
Dominican Republic	1.08 (0.96-1.22)	**1.31 (1.01-1.69)**	1.03 (0.90-1.18)	0.93 (0.84-1.03)	0.90 (0.63-1.30)
Peru urban	0.97 (0.86-1.10)	1.07 (0.83-1.39)	0.95 (0.82-1.10)	0.92 (0.82-1.02)	0.81 (0.56-1.17)
Peru rural	1.18 (0.95-1.48)	0.74 (0.45-1.21)	0.99 (0.76-1.30)	1.06 (0.86-1.29)	1.73 (0.92-3.26)
Venezuela	**1.21 (1.08-1.35)**	1.09 (0.89-1.34)	0.94 (0.82-1.08)	1.02 (0.92-1.14)	1.18 (0.81-1.73)
Mexico urban	1.08 (0.90-1.29)	0.74 (0.50-1.09)	1.01 (0.84-1.22)	1.06 (0.88-1.27)	1.00 (0.56-1.77)
Mexico rural	1.01 (0.85-1.19)	**1.50 (1.10-2.06)**	1.00 (0.81-1.23)	**0.84 (0.73-0.98)**	0.57 (0.31-1.05)
China urban	0.99 (0.82-1.18)	0.83 (0.62-1.11)	**1.20 (1.02-1.40)**	1.00 (0.76-1.33)	2.48 (0.92-6.68)
Puerto Rico	1.05 (0.94-1.19)	1.10 (0.88-1.39)	**0.88 (0.79-0.99)**	**1.16 (1.03-1.31)**	1.31 (0.92-1.89)
Pooled fixed effect	**1.07 (1.02-1.12)**	0.99 (0.90-1.09)	0.98 (0.93-1.03)	0.98 (0.94-1.03)	1.06 (0.91-1.23)
Higgins I^2(%)	17 (0-59)	54 (3-78)	41 (0-73)	54 (3-78)	42 (0-73)

1. adjusted odds ratios and their 95% confidence intervals, derived from multivariable logistic regression
2. adjusted for other sociodemographic characteristics (age, gender, and education) and number of declines in intrinsic capacity
3. adjusted for age, gender, and education

Conclusions – Attrition in the dependence cohort
Attrition in the dependence cohort is both more extensive, and more complex that in the mortality cohort. Overall one-fifth of cohort participants could not be included in the final analysis of predictors of incident dependence, since the outcome had not been recorded. The most common reason was loss to follow-up, since the outcome was determined on the majority of those who had died, through post-mortem verbal autopsy. While LTFU was consistently associated with older age, this association was independent of DIC (which was generally not associated with LTFU), and hence must have been mediated through other age related-factors. There was a suggestion that men and those with fewer DIC might be more likely to be LTFU in rural Mexico, and that those with less education and more DIC might be more likely to be LTFU in Puerto Rico. Local factors may possibly have played a part. Patterns of association between DIC, frailty and incident dependence seem similar in these sites to other sites (main paper, Table 5). Attrition bias in the estimation of these effects cannot be positively excluded. The direction would be a matter of speculation. Given the

broad reassurance that attrition is generally random, in most sites, with respect to hypothesised exposures, it seems unlikely that attrition bias will have substantially accounted for observed associations, so as to lead to erroneous conclusions.

References

1. Ferri CP, Acosta D, Guerra M, Huang Y, Llibre-Rodriguez JJ, Salas A, et al. Socioeconomic factors and all cause and cause-specific mortality among older people in Latin America, India, and China: a population-based cohort study. PLoSMed. 2012 Feb;9(1549-1676 (Electronic)):e1001179.

2. Jotheeswaran AT, Bryce R, Prina M, Acosta D, Ferri CP, Guerra M, et al. Frailty and the prediction of dependence and mortality in low- and middle-income countries: a 10/66 population-based cohort study. BMCMed. 2015 Jun 10;13:138. doi: 10.1186/s12916-015-0378-4.:138–0378.

3. Hall KS, Gao S, Emsley CL, Ogunniyi AO, Morgan O, Hendrie HC. Community screening interview for dementia (CSI ’D’); performance in five disparate study sites. Int J Geriatr Psychiatry. 2000;15:521–31.

4. Bao J, Chua K-C, Prina M, Prince M. Multimorbidity and care dependence in older adults: a longitudinal analysis of findings from the 10/66 study. BMC Public Health. 2019 May 16;19(1):585.
