# Supplementary material for: Intrinsic capacity and its associations with incident dependence and mortality in 10/66 Dementia Research Group studies in Latin America, India, and China: A population-based cohort study
Source: PLoS Med. 2021 Sep 14;18(9):e1003097. doi: 10.1371/journal.pmed.1003097 (PMC8439485; doi:10.1371/journal.pmed.1003097)
Supplement: S1 Checklist — (PDF) [file pmed.1003097.s003.pdf]

S1 CHECKLIST - STROBE Statement—Checklist of items that should be included in reports of ***cohort studies***

	Item No	Recommendation	Page No
**Title and abstract**	1	(*a*) Indicate the study's design with a commonly used term in the title or the abstract<br>(*b*) Provide in the abstract an informative and balanced summary of what was done and what was found	'Population-based cohort study' included in the title<br>Abstract, paragraph 2 Methods and Findings
**Introduction**			
Background/rationale	2	Explain the scientific background and rationale for the investigation being reported	Introduction - paragraphs 1-3
Objectives	3	State specific objectives, including any prespecified hypotheses	Introduction - paragraph 4
**Methods**			
Study design	4	Present key elements of study design early in the paper	Methods - Paragraph 1 (settings and study design)
Setting	5	Describe the setting, locations, and relevant dates, including periods of recruitment, exposure, follow-up, and data collection	Methods - Paragraph 1 (settings and study design)
Participants	6	(*a*) Give the eligibility criteria, and the sources and methods of selection of participants. Describe methods of follow-up<br>(*b*) For matched studies, give matching criteria and number of exposed and unexposed	Methods - Paragraph 1 (settings and study design)<br>Not applicable
Variables	7	Clearly define all outcomes, exposures, predictors, potential confounders, and effect modifiers. Give diagnostic criteria, if applicable	Methods - Paragraphs 2-9 (Measures)
Data sources/ measurement	8*	For each variable of interest, give sources of data and details of methods of assessment (measurement). Describe comparability of assessment methods if there is more than one group	Methods - Paragraphs 2-9 (Measures), and S2 Working Paper
Bias	9	Describe any efforts to address potential sources of bias	Methods - Paragraph 14 (Analysis) and S3 Supporting Information
Study size	10	Explain how the study size was arrived at	Methods - Paragraph 1 (Settings and study design)<br>[Not strictly relevant as a secondary data analysis, but we provide a link to the original sample size determination, through the referenced Cohort Profile publication (ref 24)]

Quantitative variables	11	Explain how quantitative variables were handled in the analyses. If applicable, describe which groupings were chosen and why	Methods - Paragraphs 10-14 (Analysis)
Statistical methods	12	(*a*) Describe all statistical methods, including those used to control for confounding<br>(*b*) Describe any methods used to examine subgroups and interactions<br>(*c*) Explain how missing data were addressed<br>(*d*) If applicable, explain how loss to follow-up was addressed<br>(*e*) Describe any sensitivity analyses	Methods - Paragraphs 10-14 (Analysis)<br>Not applicable<br>Methods - Paragraph 14 (Analysis) and S3 Supporting Information<br>Methods - Paragraph 14 (Analysis) and S3 Supporting Information<br>Not applicable
**Results**			
Participants	13*	(a) Report numbers of individuals at each stage of study—eg numbers potentially eligible, examined for eligibility, confirmed eligible, included in the study, completing follow-up, and analysed<br>(b) Give reasons for non-participation at each stage<br>(c) Consider use of a flow diagram	Results - Paragraph 1 (sample characteristics), Paragraph 4 and 5 (predictors of incident dependence), paragraph 9 (predictors of incident dependence) and S3 Supporting Information (by site, and exposure category)<br>Results - Paragraph 4 and 5 (predictors of incident dependence), paragraph 9 (predictors of incident dependence) and S3 Supporting Information<br>S3 Supporting Information
Descriptive data	14*	(a) Give characteristics of study participants (eg demographic, clinical, social) and information on exposures and potential confounders<br>(b) Indicate number of participants with missing data for each variable of interest<br>(c) Summarise follow-up time (eg, average and total amount)	Results - Paragraph 1 (sample characteristics) and Tables 1 and Table 3 (by exposure category)<br>Results - Table 1 and Table 2<br>Results - Paragraph 4 (predictors of incident dependence), and Paragraph 9 (predictors of incident dependence)
Outcome data	15*	Report numbers of outcome events or summary measures over time	Results - Paragraph 4 (predictors of incident dependence), and Paragraph 9 (predictors of incident dependence)

Main results	16	(*a*) Give unadjusted estimates and, if applicable, confounder-adjusted estimates and their precision (eg, 95% confidence interval). Make clear which confounders were adjusted for and why they were included<br>(*b*) Report category boundaries when continuous variables were categorized<br>(*c*) If relevant, consider translating estimates of relative risk into absolute risk for a meaningful time period	Results - Table 3-6 and S4 Table<br>Results - Table 3-6 and S4 Table<br>Not done
Other analyses	17	Report other analyses done—eg analyses of subgroups and interactions, and sensitivity analyses	Not applicable
**Discussion**			
Key results	18	Summarise key results with reference to study objectives	Discussion – Paragraph 1
Limitations	19	Discuss limitations of the study, taking into account sources of potential bias or imprecision. Discuss both direction and magnitude of any potential bias	Discussion – Paragraph 3
Interpretation	20	Give a cautious overall interpretation of results considering objectives, limitations, multiplicity of analyses, results from similar studies, and other relevant evidence	Discussion – Paragraphs 3-7
Generalisability	21	Discuss the generalisability (external validity) of the study results	Discussion – Paragraph 4, 6 and 7
**Other information**			
Funding	22	Give the source of funding and the role of the funders for the present study and, if applicable, for the original study on which the present article is based	Information provided in the course of submission

*Give information separately for exposed and unexposed groups.

Note: An Explanation and Elaboration article discusses each checklist item and gives methodological background and published examples of transparent reporting. The STROBE checklist is best used in conjunction with this article (freely available on the Web sites of PLoS Medicine at http://www.plosmedicine.org/, Annals of Internal Medicine at http://www.annals.org/, and Epidemiology at http://www.epidem.com/). Information on the STROBE Initiative is available at http://www.strobe-statement.org.
