# Supplementary material for: Intrinsic capacity and its associations with incident dependence and mortality in 10/66 Dementia Research Group studies in Latin America, India, and China: A population-based cohort study
Source: PLoS Med. 2021 Sep 14;18(9):e1003097. doi: 10.1371/journal.pmed.1003097 (PMC8439485; doi:10.1371/journal.pmed.1003097)
Supplement: S1 Table — (PDF) [file pmed.1003097.s004.pdf]

S1 Table

Adjusted associations[1] of declines in intrinsic capacity (DIC), and DIC stratified by frailty status, with mortality, by site

	No DIC n=4272	DIC n=9,664	DIC, with no frailty or needs for care n=3,573	DIC and pre-frail n=3.454	DIC and frail n=1,414	DIC and needs for care n=1,223
Cuba	1 (ref)	1.61 (1.29-2.02)	1.13 (0.87-1.48)	1.53 (1.18-1.98)	2.23 (1.66-2.99)	4.16 (3.09-5.60)
Dominican Republic	1 (ref)	1.36 (0.99-1.85)	1.09 (0.77-1.54)	1.08 (0.76-1.54)	1.78 (1.24-2.57)	3.12 (2.15-4.54)
Puerto Rico	1 (ref)	2.09 (1.54-2.82)	2.02 (1.42-2.88)	1.54 (1.07-2.23)	2.21 (1.45-3.36)	3.89 (2.61-5.80)
Peru Urban	1 (ref)	1.87 (0.98-3.56)	1.07 (0.47-2.41)	1.09 (0.50-2.35)	1.93 (0.91-4.12)	7.40 (3.54-15.45)
Peru Rural	1 (ref)	0.97 (0.49-1.90)	0.81 (0.34-1.96)	0.80 (0.36-1.78)	0.90 (0.36-2.18)	3.93 (1.55-9.97)
Venezuela	1 (ref)	2.13 (1.43-3.17)	1.62 (1.01-2.63)	1.75 (1.10-2.79)	3.81 (2.26-6.42)	3.96 (2.31-6.79)
Mexico Urban	1 (ref)	1.85 (1.10-3.10)	1.52 (0.77-2.99)	1.19 (0.64-2.23)	2.93 (1.53-5.61)	3.87 (1.93-7.77)
Mexico Rural	1 (ref)	1.69 (0.97-2.96)	1.21 (0.62-2.33)	1.45 (0.75-2.82)	2.12 (1.03-4.39)	4.44 (2.17-9.08)
China Urban	1 (ref)	1.55 (1.17-2.05)	0.91 (0.60-1.39)	0.81 (0.40-1.60)	1.58 (0.60-4.15)	2.77 (1.98-3.88)
China Rural	1 (ref)	1.56 (1.14-2.12)	1.31 (0.94-1.82)	1.64 (1.11-2.41)	2.23 (1.30-3.81)	4.26 (2.77-6.53)
India Urban	1 (ref)	1.83 (1.15-2.91)	1.52 (0.88-2.60)	1.97 (1.19-3.25)	2.21 (1.23-3.95)	1.62 (0.66-3.96)
Pooled effect size	1 (ref)	1.66 (1.49-1.85)	1.27 (1.12-1.45)	1.42 (1.24-1.63)	2.20 (1.89-2.56)	3.92 (3.43-4.49)
Heterogeneity - Higgins I^2% (95% CIs)	1 (ref)	0 (0-60)	39 (0-70)	1 (0-61)	0 (0-60)	33 (0-67)

1. From a Cox's proportional hazards regression, controlling for age, gender, and education, generating adjusted hazard ratios and their 95% confidence intervals
