# Supplementary material for: Intrinsic capacity and its associations with incident dependence and mortality in 10/66 Dementia Research Group studies in Latin America, India, and China: A population-based cohort study
Source: PLoS Med. 2021 Sep 14;18(9):e1003097. doi: 10.1371/journal.pmed.1003097 (PMC8439485; doi:10.1371/journal.pmed.1003097)
Supplement: S1 Working Paper — (PDF) [file pmed.1003097.s005.pdf]

S1 WORKING PAPER
DEPENDENCE (NEEDS FOR CARE) IN THE 10/66 DEMENTIA RESEARCH GROUP STUDIES

The 10/66 Dementia Research Group
King's College London
January 2020

SUMMARY
In this working paper we set out to describe and justify the definition of dependence applied in the 10/66 Dementia Research Group studies, the assessment procedures, and measurement approach. We summarise the research that we have conducted into this outcome. We provide evidence for the construct (concurrent and predictive) validity of the measure.

Dependence relates to needs for care, regardless of whether these are routinely met. Needs for care should arise from health conditions, and be adjudged as exceptional with respect to family and sociocultural norms. We used a semi-structured assessment approach, based upon open-ended scoping questions, followed by a detailed structured assessment of care arrangements. Interviewer ratings of care dependence were ultimately made in the light of all information gathered and observations made, formally and informally, throughout the survey assessment.

We have reported on the prevalence and correlates of dependence across 10/66 catchment area sites. Prevalence was higher in older age groups, among men, and those with less education. Disorders of the mind and brain (dementia, stroke and depression) were among the leading contributors. Age-standardised prevalence was generally lower than has been reported in high income country settings, particularly in our rural and less developed sites. This may be explained by difficulty in distinguishing exceptional needs, when most older people receive support. Survival with needs for care may also be short in these settings. In prospective cohort studies we have demonstrated robust independent associations between both frailty and multimorbidity at baseline, and the incidence of dependence.

We could not identify a suitable validated measure when planning our research, and there is still no accepted 'gold standard' for the settings in which we work. We have therefore explored construct validity, which is strongly supported by the pattern of associations observed, with informant reported average daily hours of care, and participant self-reported disability (the WHODAS 2.0 Disability Assessment Scale). An important limitation, given the nature of the assessment and rating procedure, is the lack of available information on inter-rater reliability of the measure.

CONTENTS

Page	Section	Table or Figure	Content
**2**	**1**		**What is dependence?**
**2**	**2**		**How was dependence assessed in the 10/66 Dementia Research Group studies?**
**2**	**3**		**How has dependence been researched in the 10/66 Dementia Research Group Studies?**
**4**	**4**		**Evidence for the validity of the 10/66 dependence measure**
**6**		Fig 1	Boxplot of average daily time (in hours) spent in assisting older person with activities of daily living, by needs of care
**7**		Fig 2	Boxplot of WHO Disability Assessment Scale (WHODAS 2.0) total scores, by needs of care
**8**		Fig 3	Box plot of distribution of WHODAS 2.0 disability scores, by site and dependence status
**9**		Table 1	Optimal cut off points, sensitivity, specificity, area under the curve and 95% confidence intervals for WHODAS 2.0 scores versus dependence by 10/66 sites
**10**		Fig 4	WHODAS Item 8 (difficulty with washing whole body), by needs for care
**10**		Fig 5	WHODAS Item 9 (difficulty with getting dressed), by needs for care
**11**		Fig 6	WHODAS Item 10 (difficulty with dealing with people you don't know), by needs for care
**11**		Fig 7	WHODAS Item 11 (difficulty with maintaining a friendship), by needs for care
**12**	**5**		References

1. What is dependence?

In the approach adopted by the 10/66 studies, dependence follows the concept postulated by Harwood and collaborators (1) who defined dependence as
"the requirement for frequent help from other person beyond that would be expected by virtue of family and social ties experienced by the person in need, in comparison to others under the same socioeconomic and cultural circumstances."
The definition of dependence applied in the 10/66 studies modifies Harwood's operationalisation of the concept by the addition of the cavil that the requirement for frequent help should be 'due to a health condition'. This would explicitly exclude from consideration, for example, the dependence that affluent people might have on paid home help. It aligns the definition of dependence with the WHODAS 2.0 disability concept (2), which refers to difficulty in completing a task, 'because of a health condition', again excluding difficulties arising from for example gender, discrimination, political oppression. The underlying premise is that disability and dependence are related constructs. The effect of this modification is that disability becomes a necessary condition for dependence to occur. However, not all people with disability are dependent – disability may arise from increased difficulty in performing a task without frequent help being required or provided.

2. How was dependence assessed in the 10/66 Dementia Research Group studies?

The choice of assessment procedure was predicated by the lack of any suitable brief structured assessment with demonstrable cross-cultural validity, and the lack of time to invest in development and validation of a new tool. Open-ended questioning, and local interviewer judgments after

orientation and training seemed to offer the best hope of incorporating local norms, and achieving cross-cultural equivalence in the ratings. To assess needs for care, the interviewer asked a series of open-ended questions to the key informant:
1. Who shares the home with the participant?
2. What kind of help does the participant need inside the home?
3. What kind of help does the participant need outside of the home?
4. Who, in the family, is available to care for the participant?
5. What help do you provide?
6. Do you help to organise care for the participant?
7. Is there anyone else in the family who is more involved in helping than you? What do they do?
8. What about friends and neighbours? What do they do?

Based on the informant's answers, and the totality of the evidence collected by the interviewer during the comprehensive survey assessment, including direct observation of the home environment and interactions with others in the household, the interviewer was then instructed to code whether the participant required:
1. no care,
2. care some of the time, or
3. care much of the time.

The coding was based upon the interviewer's perception of needs for care, independent of whether these were routinely met. One significant limitation was that we did not strictly operationalise the distinction between care 'some of the time' and care 'much of the time'. However, for the purposes of almost all analyses conducted and described in this working paper, dependence is treated as a dichotomous variable, any needs for care versus no needs for care, hence the impact of the difficulty in rating some versus much care is minimised.

3. How has dependence been researched in the 10/66 Dementia Research Group Studies?
An understanding of the determinants of dependence is an essential prerequisite for prevention, long-term care policymaking and planning. Older people are likely to have multiple health conditions – chronic physical diseases affecting different organ systems, coexisting with mental and cognitive disorders - interacting in complex ways to create difficulties in performing important tasks and activities, and in determining needs for care.

We carried out descriptive research out using data from the cross-sectional baseline surveys in Latin America, China, India and Nigeria. Detailed accounts of the epidemiology of dependence in the Dominican Republic (3), and Nigeria (4), were followed by a multi-centre analysis of the prevalence and correlates of dependence in the Latin America, China and India sites (5). In this study, the prevalence of dependence increased sharply with increasing age, was higher among women than men, and among those with least education. Overall prevalence for those aged 65 years and over varied among 10/66 sites, from 2.9% in urban India to 15.7% in urban China. In most other sites, the indirectly standardized prevalence of dependence was one half to three quarters of that reported in the USA National Long Term Care Survey (6). The Standardised Morbidity Ratios for urban India (21.5), rural Peru (27.5) and rural China (38.2) were strikingly low, while that for urban China (98.3) indicated a similar prevalence to the USA reference population. The tendency towards a lower prevalence in rural and less developed sites was accentuated after directly standardizing for demographic status. Dementia emerged as by far the leading independent chronic disease contributor to dependence. Limb weakness, stroke, depression, eyesight problems and arthritis made more modest contributions. Effect sizes for associations with dependence were generally larger than those for the same associations with disability, previously reported in the same samples (7).

In addition to the USA National Long Term Care surveys (6) (reporting 17.1% disabled in one or more activities of daily living, or living in a care home) our estimates of the crude prevalence of dependence among those aged 65 years and over in low- and middle-income countries (LMIC) were generally lower than those reported in other previous population-based studies of older people in high income countries; in England and Wales (8) (15.7% with significant disability among whom 86% had dependence needs), Scotland (9) (15% with short interval dependence), Spain (10) (15.5% with dependence in one or more of seven ADLs), France (11) (12.4% confined to home or bed). Notably, only one of these surveys had assessed dependence directly (9), the others inferring care dependence from an assessment of disability/ functioning.

A relatively lower age-specific prevalence of dependence in less developed and rural LMIC settings may be explained by a lower prevalence of chronic disease. Alternatively, given that prevalence is the product of incidence and duration, it may be that survival in a state of dependence is much shorter in LMIC settings. Another possible explanation in rural sites is that most older people live with their children and are routinely provided with support for both core and instrumental activities of daily living, therefore it may be difficult to identify the onset of 'the need for frequent human help or care beyond that habitually required by a healthy adult'. It has been reported that urbanisation in Egypt has contributed to a growing awareness of unmet needs for care among older people because poor immigrant families tend to live in slum districts and need to work to maximise household income, leaving dependent older relatives without assistance (12).

After completing the incidence wave, a follow-up in Latin America and China 3-5 years after the baseline survey, we have also reported on the associations between frailty (13) and multimorbidity (14) at baseline, and the subsequent incidence of dependence.

In the frailty cohort analysis (13), both the Fried and Strawbridge frailty phenotypes at baseline predicted the onset of dependence and mortality, even adjusting for chronic diseases and disability, with little heterogeneity of effect among sites. However, population attributable fractions summarising aetiologic force were highest for the aggregate effect of the individual frailty indicators, as opposed to either the number of indicators or the dichotomised frailty phenotypes. We concluded that simply assessed frailty indicators identify older people at risk of dependence and mortality, beyond information provided by chronic disease diagnoses and disability.

In the multimorbidity cohort analysis (14), each unit increase in multimorbidity count at baseline increased the cumulative risk of incident dependence by 20% in the fully adjusted model. Age was the only variable to confound this relationship. Physical multimorbidity was associated with only a modest increased risk of care dependence. Dementia, depression and anxiety were independently associated with incident care dependence at every level of physical multimorbidity, and depression and anxiety attenuated the effect of physical multimorbidity. We concluded that multimorbidity consistently predicts care dependence with little variation between countries, and that mental and cognitive disorders independently increase the risk of care dependence.

4. Evidence for the validity of the 10/66 dependence measure

We adopted a pragmatic approach for the assessment of dependence, in the absence of previous research in LMIC, given the difficulties of developing a more structured assessment with demonstrable validity across many different countries and cultures. Other studies have inferred dependence from limitations in core activities of daily living, usually ascertained from the participant. Our approach was more direct, and the ascertainment of needs for care from the care provider, rather than the care recipient may have avoided under-reporting due to social desirability or cognitive impairment.

Nevertheless, important questions remain regarding the validity of our measurement approach. Since there is no established 'gold standard' measure of care dependence in general, and certainly not one applicable across a range of LMIC cultures and health and social care systems, criterion validation is not an option. The aforementioned cross-sectional associations between chronic diseases prone to lead to needs for care (5), and between frailty (13), multimorbidity (14) and incident dependence support concurrent validity. We have also carried out, but not previously reported, further detailed analyses of concurrent validity against two somewhat independent measures of constructs that one would expect to be positively correlated with a valid measure of needs for care

1. Average daily time spent in assisting the older person with activities of daily living. This was assessed from an informant who was the main caregiver for the older person. We enquired after time spent assisting with seven activities; communication, transport, dressing, eating, grooming, toileting, and bathing
2. Disability, as assessed using the WHO Disability Assessment Scale (WHODAS 2.0) (2). The respondent for this assessment was the older person, unless they were too impaired to provide reliable responses, in which case a proxy version was administered to a key informant, usually a co-resident family member.

Time spent assisting the older person with activities of daily living should be positively correlated with dependence, but not perfectly so, since we have conceptualised dependence as needs for care rather than provision of care. Therefore, an older person could still be correctly rated as having needs for care, even if those needs were not routinely being met. Conversely, an older person may be receiving assistance, when they might be correctly adjudged to be, in principle, independent. The distribution of average daily hours spent in assisting the older person with activities of daily living is summarised in the box plot in Figure 1 below stratified by needs for care.

Figure 1
Boxplot of average daily time (in hours) spent in assisting older person with activities of daily living, by needs of care

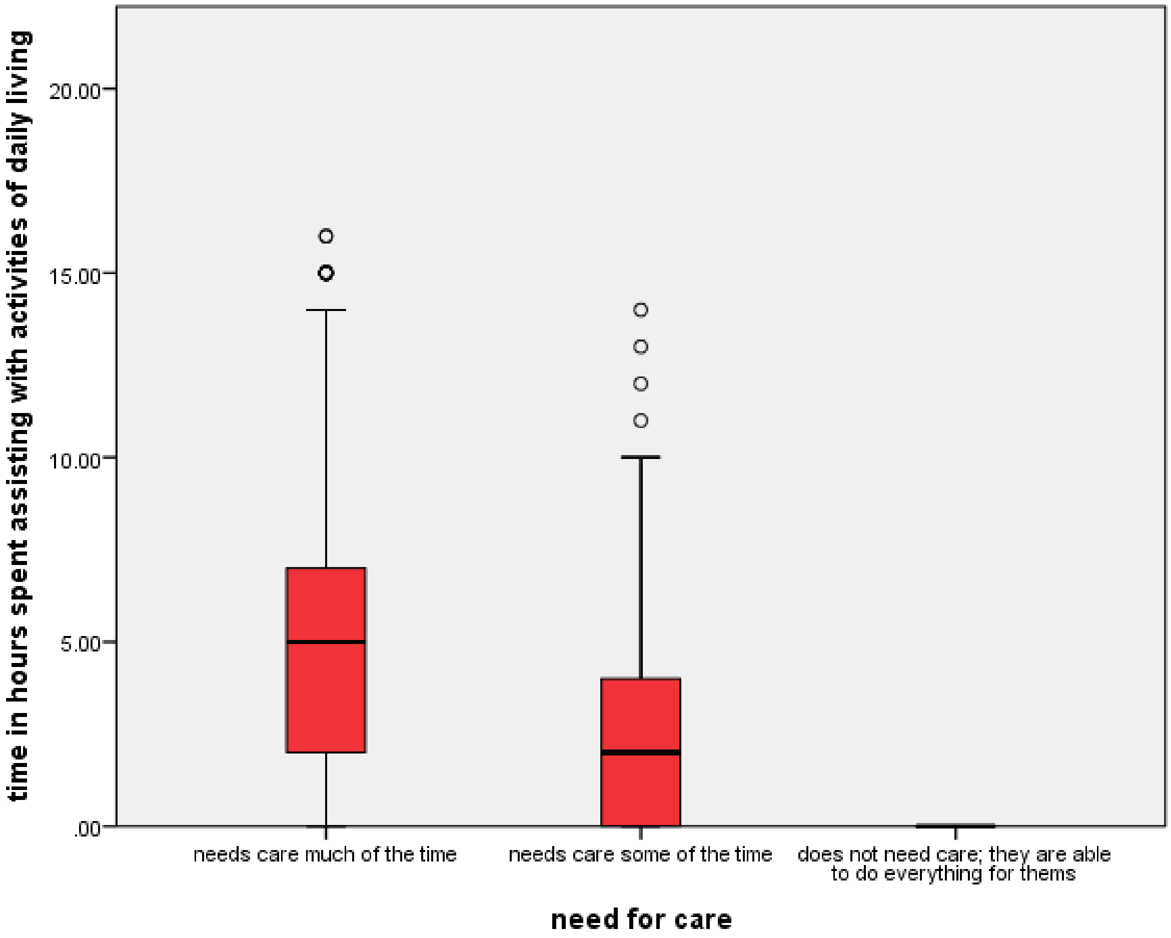

Consistent with our hypothesis, the distribution of hours spent assisting with ADL is clearly very different between the three groups with a median of zero hours for those rated as not needing care, 2.5 hours for those needing care some of the time, and five hours for those needing care much of the time.

As previously discussed, the concepts of disability and dependence are closely inter-related, as are the measures and indicators that are used to assess them. Dependence is, in general, a consequence of disability, although not all those with disability may need care. Some people with disabilities may simply have trouble in carrying out a task, without loss of independence in its performance; perhaps taking longer over it, using mechanical aids, or experiencing discomfort or inconvenience. 'High level' tasks (e.g. playing bridge for someone with early dementia, or playing golf for someone with osteoarthritis of the hip) may no longer be able to be completed, and these and other demanding activities may be forgone, without the person needing care. Severe disability in core activities of daily living has been widely used as a proxy indicator of dependence (needs of care) (1). We can therefore hypothesise that being dependent according to our definition (having any needs for care) as rated by the interviewer should be associated with higher scores on the WHODAS 2.0 Disability Assessment Scale.
At baseline, across the 13 catchment area sites that contributed to the prevalence wave survey needs for care were rated on 16,486 participants. Of these 14,902 were rated as not needing care, 737 (4.5%) as needing care some of the time, and 847 (5.1%) as needing care much of the time.

As indicated in the boxplot (Figure 2) the distribution of WHODAS 2.0 scores was distinctively different between those not needing care, those needing care some of the time, and those needing care much of the time.

Figure 2
Boxplot of WHO Disability Assessment Scale (WHODAS 2.0) total scores, by needs of care

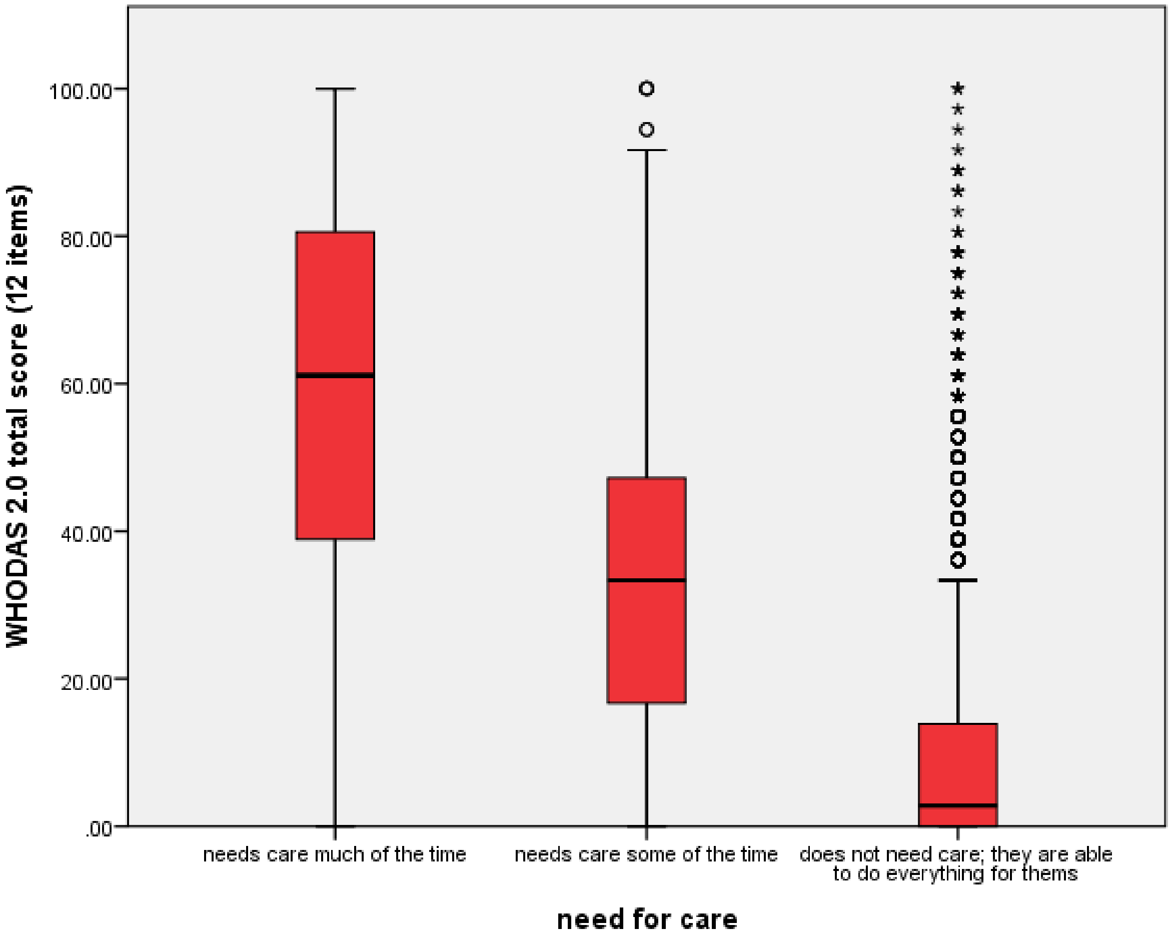

o = outliers; * = extreme values

While the distribution of scores among those who were dependent and independent varied among sites (Figure 3), with, for example, generally higher scores in rural India and lower scores in urban Mexico, in every site there was, as hypothesised, a clear distinction between score distributions for those who were rated as dependent, and those rated as independent.

Figure 3 - Box plot of distribution of WHODAS 2.0 disability scores, by site and dependence status

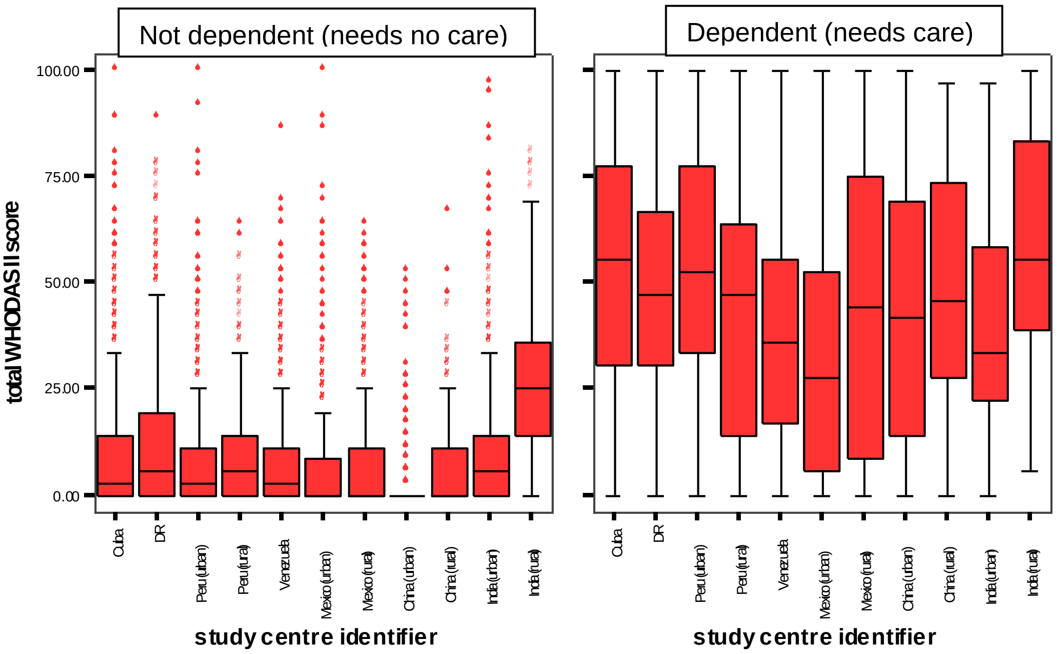

o = outliers; * = extreme values

As a measure of correspondence of the WHODAS 2.0 score and the dependence criterion, we used receiver operating characteristic (ROC) analysis treating dependence (needing any care) as the criterion, and the WHODAS 2.0 as the test score. The area under the receiver operating characteristic curve (AUROC), with 95% confidence intervals was calculated for the WHODAS 2.0 scores with dependence (any need of care) as the criterion, for each 10/66 site. ROC curves plot sensitivity versus 1-specificity for every possible cut-point on the test measure. They are used both to convey information regarding the overall predictiveness of a measure, and to help identify the optimal cut-point that simultaneously maximises sensitivity and specificity. The area under the curve is the coefficient used to quantify the ability of the test scale to discriminate accurately between positives and negatives. For a useless test, the area under the curve is 0.5. A perfect test (one that has zero false positives and zero false negatives) has an AUROC of 1.00 .

The AUROC varied from 0.78 in urban Mexico to 0.95 in rural China suggesting that the performance of the WHODAS in discriminating between those not needing care and those needing any care is generally good (Table 1). The optimal cut-off points ranged by site from 6.9 to 31.9, with a median of 20.8.

Table 1 – Optimal cut off points, sensitivity, specificity, area under the curve and 95% confidence intervals for WHODAS 2.0 scores versus dependence by 10/66 sites

	AUC (95% CI)	Optimal cut point	Sensitivity	Specificity	Youden's Index
**Cuba**	0.91 (0.88-0.93)	20.8	0.86	0.84	0.70
**DR**	0.89 (0.87-0.92)	23.6	0.84	0.79	0.63
**Peru urban**	0.92 (0.89-0.95)	20.8	0.85	0.85	0.70
**Peru rural**	0.86 (0.77-0.94)	31.9	0.65	0.95	0.60
**Venezuela**	0.87 (0.84-0.91)	12.5	0.83	0.76	0.59
**Mexico urban**	0.78 (0.74-0.84)	9.7	0.71	0.76	0.47
**Mexico rural**	0.82 (0.76-0.88)	20.8	0.69	0.85	0.54
**China urban**	0.93 (0.91-0.96)	6.9	0.86	0.93	0.79
**China rural**	0.95 (0.92-0.98)	20.8	0.86	0.93	0.79
**India urban**	0.82 (0.72-0.92)	18.1	0.83	0.82	0.65
**India rural**	0.86 (0.82-0.92)	31.9	0.85	0.69	0.54

We have previously shown that the WHODAS 2.0 is a strong hierarchical measurement scale, and that the item difficulties are largely invariant between sites (15). Therefore, those with low WHODAS 2.0 scores will have generally endorsed only low-item difficulty items whereas those with high scores will also have endorsed high-item difficulty items. Since high WHODAS 2.0 scores are associated with dependence, then it should be, by extension, endorsement of the high item difficulty items that distinguish those with and without dependence. The four highest item difficulty items, across all sites, are dealing with people you do not know, maintaining a friendship, washing the whole body, and getting dressed (15) – difficulties in these areas are particularly likely to be associated with needs for care. Accordingly, we hypothesised that self-reported responses to the level of difficulty (none, mild, moderate, severe, extreme/ cannot do) encountered in the performance of these tasks would discriminate particularly effectively between those rated as having no needs for care, needs for care some of the time, and needs for care much of the time.

The results of these analyses are summarised in bar charts (Figures 4 to 7). The pattern of findings is clear and consistent across all four items, as predicted by our hypothesis. Almost all of those rated as having no needs for care report no difficulties, and where difficulties are reported they are mild or moderate. Only a quarter to a half of those rated as having needs for care much of the time report no difficulty, while a substantial proportion report severe or extreme difficulty.

Figure 4 WHODAS Item 8 (difficulty with washing whole body), by needs for care

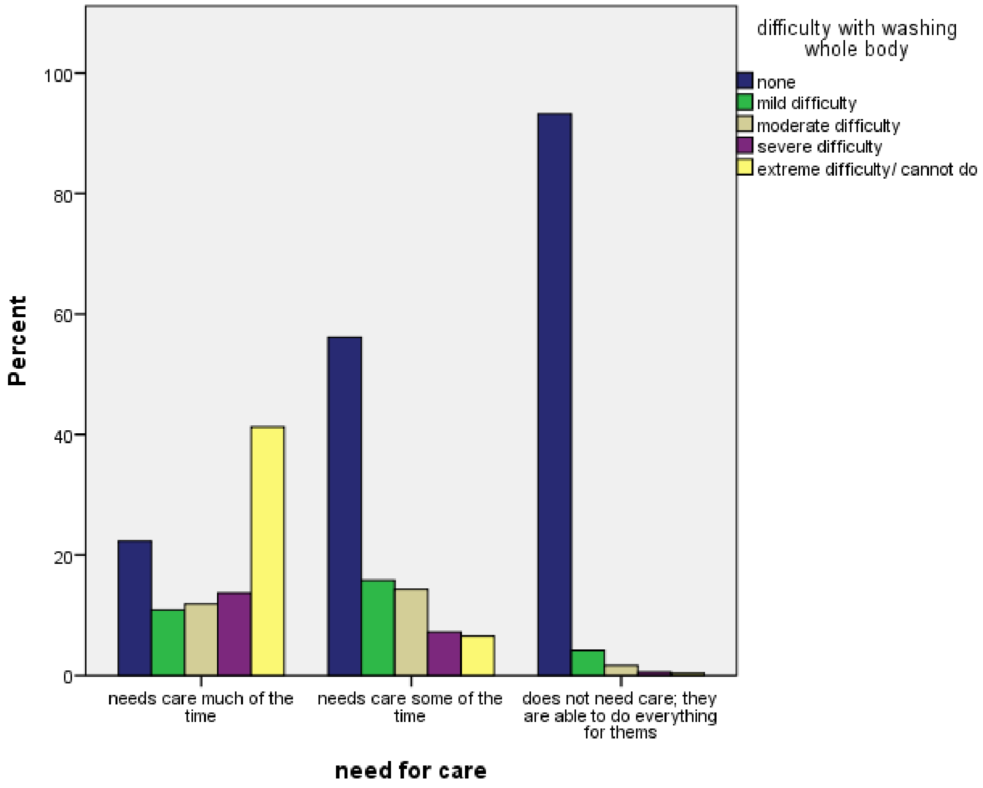

Figure 5 WHODAS Item 9 (difficulty with getting dressed), by needs for care

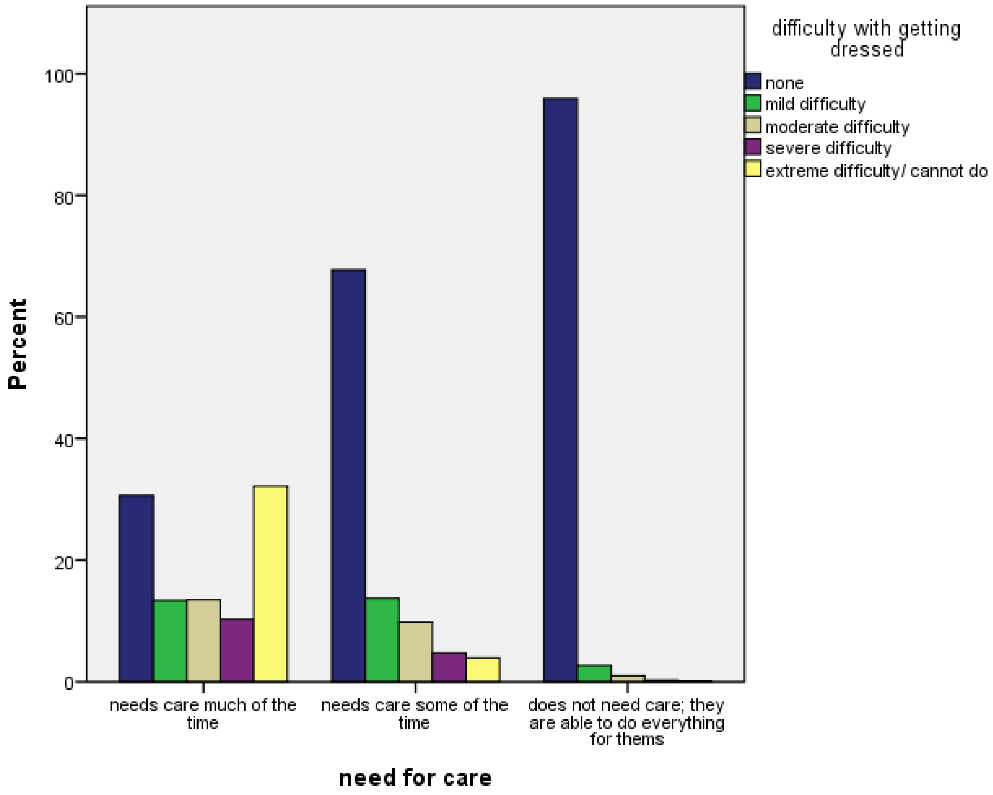

Figure 6 WHODAS Item 10 (difficulty with dealing with people you don't know), by needs for care

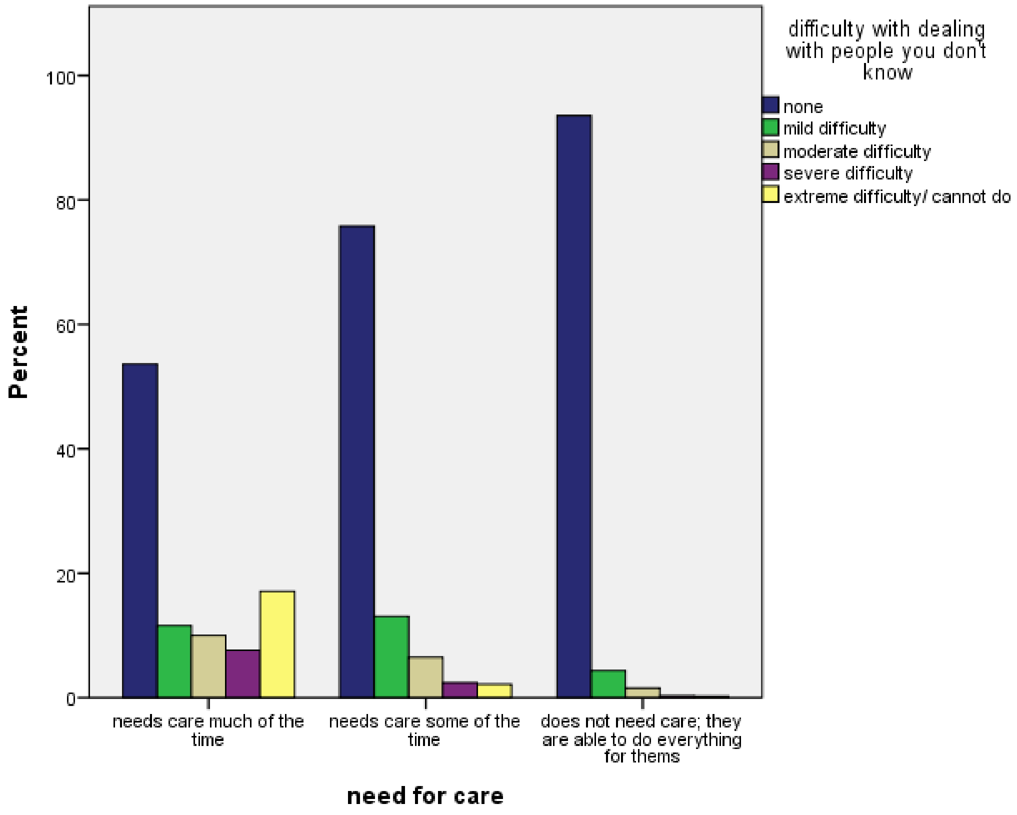

Figure 7 WHODAS Item 11 (difficulty with maintaining a friendship), by needs for care

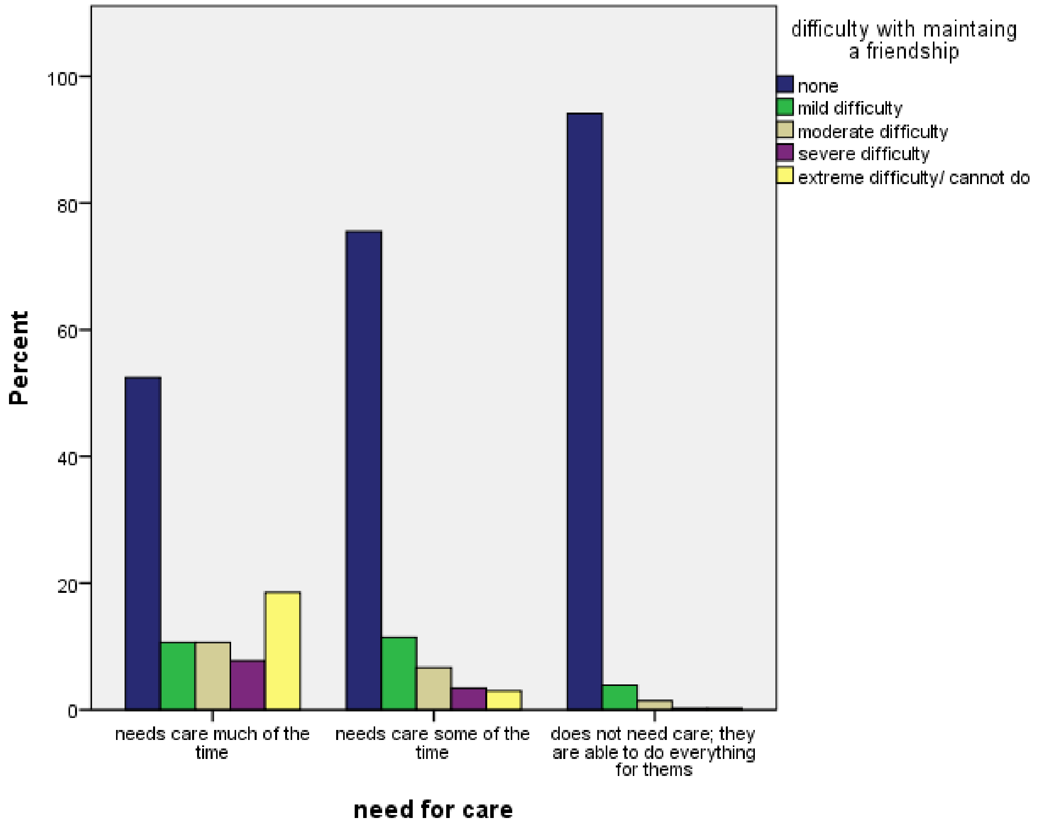

REFERENCES

1. Harwood RH, Sayer AA, Hirschfeld M. Current and future worldwide prevalence of dependency, its relationship to total population, and dependency ratios. BullWorld Health Organ. 2004 Apr;82(4):251–8.

2. World Health Organization. Measuring Health and Disability. Manual for WHO Disability Assessment Schedule (WHODAS 2.0). Geneva: WHO Press; 2010.

3. Acosta D, Rottbeck R, Rodriguez G, Ferri CP, Prince MJ. The epidemiology of dependency among urban-dwelling older people in the Dominican Republic; a cross-sectional survey. BMCPublic Health. 2008 Aug 13;8(1471-2458 (Electronic)):285.

4. Uwakwe R, Ibeh CC, Modebe AI, Bo E, Ezeama N, Njelita I, et al. The Epidemiology of Dependence in Older People in Nigeria: Prevalence, Determinants, Informal Care, and Health Service Utilization. A 10/66 Dementia Research Group Cross-Sectional Survey. JAmGeriatrSoc. 2009 Aug 13;(1532-5415 (Electronic)).

5. Sousa RM, Ferri CP, Acosta D, Guerra M, Huang Y, Ks J, et al. The contribution of chronic diseases to the prevalence of dependence among older people in Latin America, China and India: a 10/66 Dementia Research Group population-based survey. BMCGeriatr. 2010 Aug 6;10(1471-2318 (Electronic)):53.

6. Manton KG, Corder L, Stallard E. Chronic disability trends in elderly United States populations: 1982-1994. ProcNatlAcadSciUSA. 1997 Mar 18;94(6):2593–8.

7. Sousa RM, Ferri CP, Acosta D, Albanese E, Guerra M, Huang Y, et al. Contribution of chronic diseases to disability in elderly people in countries with low and middle incomes: a 10/66 Dementia Research Group population-based survey. Lancet. 2009 Nov 28;374(1474-547X (Electronic)):1821–30.

8. Melzer D, McWilliams B, Brayne C, Johnson T, Bond J. Profile of disability in elderly people: estimates from a longitudinal population study. BMJ. 1999 Apr 24;318(7191):1108–11.

9. Bond J, Carstairs V. Services for the elderly: a survey of the characteristics and needs of a population of 5000 older people. London: HMSO; 1982.

10. Beland F, Zunzunegui MV. Predictors of functional status in older people living at home. Age Ageing. 1999 Mar;28(2):153–9.

11. Leibovici D, Curtis S, Ritchie K. The application of disability data from epidemiological surveys to the development of indicators of service needs for dependent elderly people. Age Ageing. 1995 Jan;24(1):14–20.

12. Boggatz T, Dassen T. Ageing, care dependency, and care for older people in Egypt: a review of the literature. J Clin Nurs. 2005 Sep;14(8B):56–63.

13. Jotheeswaran AT, Bryce R, Prina M, Acosta D, Ferri CP, Guerra M, et al. Frailty and the prediction of dependence and mortality in low- and middle-income countries: a 10/66 population-based cohort study. BMCMed. 2015 Jun 10;13:138. doi: 10.1186/s12916-015-0378-4.:138–0378.

14. Bao J, Chua K-C, Prina M, Prince M. Multimorbidity and care dependence in older adults: a longitudinal analysis of findings from the 10/66 study. BMC Public Health. 2019 May 16;19(1):585.

15. Sousa RM, Dewey ME, Acosta D, Jotheeswaran AT, Castro-Costa E, Ferri CP, et al. Measuring disability across cultures--the psychometric properties of the WHODAS II in older people from seven low- and middle-income countries. The 10/66 Dementia Research Group population-based survey. IntJMethods PsychiatrRes. 2010 Mar;19(1049-8931 (Print)):1–17.
